# Correction: How is *Etuaptmumk*/Two-Eyed Seeing characterized in Indigenous health research? A scoping review

**DOI:** 10.1371/journal.pone.0307249

**Published:** 2024-07-11

**Authors:** Sophie I. G. Roher, Ziwa Yu, Debbie H. Martin, Anita C. Benoit

Fig 2 is incorrect. The authors have provided a corrected version here.

**Fig 2:**
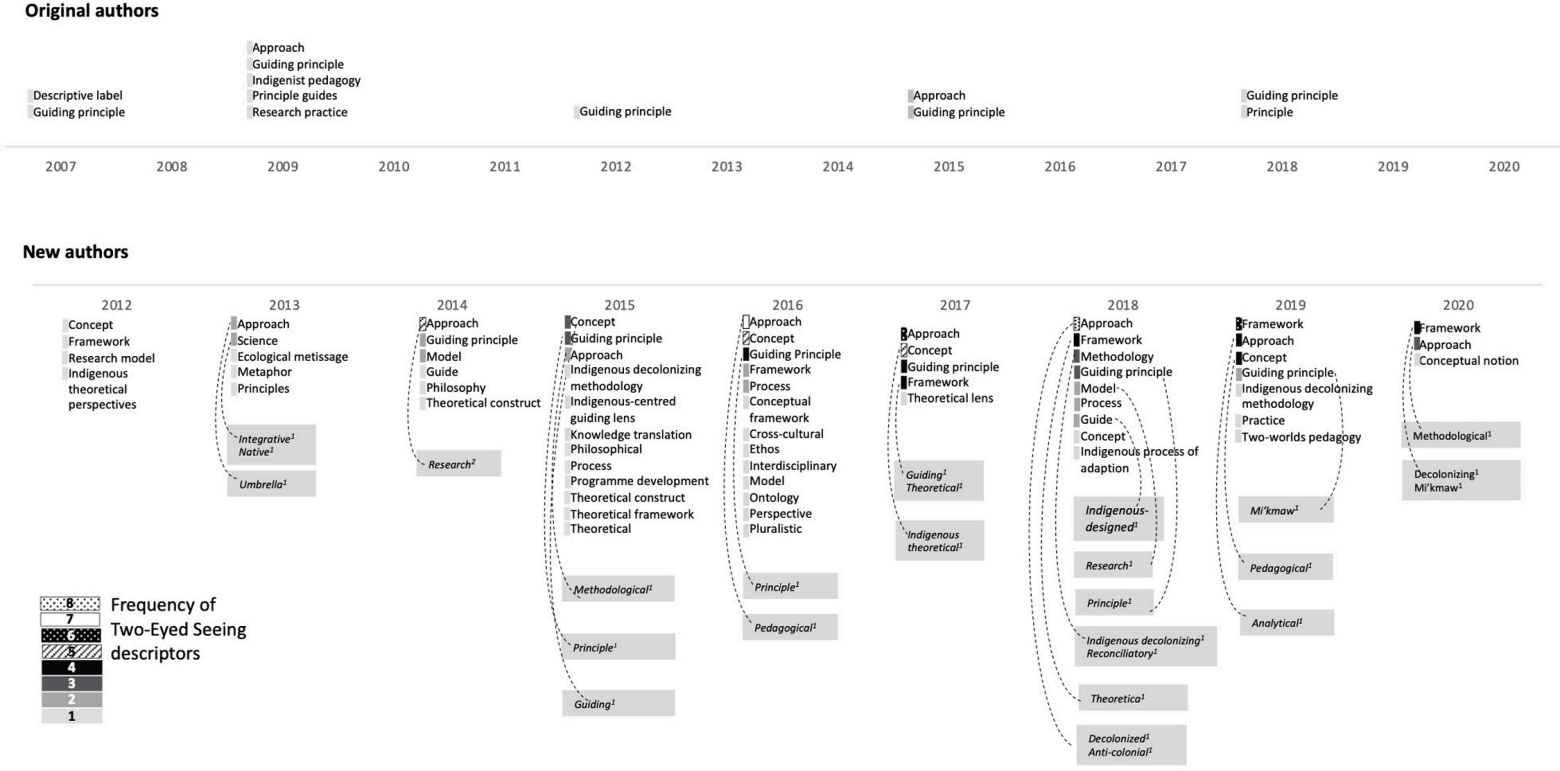
Two-Eyed Seeing terminologies used by original and new authors over time. Dotted lines represent qualifiers or extensions of the descriptors that are part of the total number.
