# Supplementary material for: How is Etuaptmumk/Two-Eyed Seeing characterized in Indigenous health research? A scoping review
Source: PLoS One. 2021 Jul 20;16(7):e0254612. doi: 10.1371/journal.pone.0254612 (PMC8291645; doi:10.1371/journal.pone.0254612)
Supplement: S1 File — Original manuscript by Elder Albert Marshall and Dr. Cheryl Bartlett in Encyclopedia of Educational Philosophy and Theory (Springer online) edited by Michael A. Peters within section “Indigenous Education in Canada” coordinated by Michelle Hogue. (PDF) [file pone.0254612.s001.pdf]

Etuaptmumk / Two-Eyed Seeing for Knowledge Gardening

forthcoming in: Encyclopedia of Educational Philosophy and Theory (Springer online) edited by Michael A. Peters within section "Indigenous Education in Canada" coordinated by Michelle Hogue
submitted: 7 February 2018

CO-AUTHOR IDENTIFICATION
Albert Marshall, Elder, Honorary Doctorate. Eskasoni, Mi'kmaw Nation.
albertdmarshall@ns.sympatico.ca
Cheryl Bartlett, CM, PhD. Cape Breton University, Sydney, Canada. cheryl_bartlett@cbu.ca

INTRODUCTION

Understandings herein have been grown over many years by the co-authors working closely together and in conjunction with others. Congruent with Indigenous relational philosophy, life context ("positionality") for the co-authors is provided. **Albert Marshall** lives in Eskasoni, Nova Scotia (NS), Canada, in Mi'kma'ki, the Traditional Territory of the Mi'kmaw Nation in eastern North America. He is a highly-respected, well-known Elder and also an ex-inmate of the Canadian residential school system. Together with his wife Elder Murdena, Albert has long worked to share understandings about Mi'kmaw language and knowledge and to encourage the "working together" of Indigenous and mainstream peoples and knowledges. Albert is the designated voice for Elders in regards environmental issues in Unama'ki - Cape Breton, NS. **Cheryl Bartlett** is of newcomer lineage (European) and grew up in Blackfoot Traditional Territory in the prairies of southern Alberta, Canada. She has lived in Sydney, NS, since 1989 and is Professor Emerita of Biology and former Tier 1 Canada Research Chair in Integrative Science at Cape Breton University (CBU). In conjunction with Mi'kmaw educators and Elders (especially Murdena and Albert Marshall), community organizations, and mainstream allies, Cheryl helped create and teach the *Toqwa'tu'kl Kjijitaqnn* / Integrative Science (TK/IS) program at CBU which sought "to bring together Indigenous and Western scientific knowledges" for university-level science education (see IS website).

This contribution was written by Cheryl using participatory narrative as appropriate within Indigenous work. Many of the understandings mentioned first grew in the above-mentioned TK/IS educational program, then spread to other science and related arenas where they continue to evolve; select examples are mentioned in "knowledge gardening".

THE MAIN CHALLENGE FOR INDIGENOUS EDUCATION IN CANADA TODAY, Elder Albert indicates, is to prepare students to walk in two worlds: their Indigenous Nation and mainstream society. Indigenous education needs to help youth understand who they are, where they come from, and how to speak their Ancestors' language. Why? In Albert's words: "Because when you force someone to abandon their ways of knowing, their ways of seeing the world, you literally destroy their Spirit and once that Spirit is destroyed it is very, very difficult to embrace anything – academically or through sports or through arts or through anything – because that person is never whole. To have a whole person, their Spirit, their physical being, their emotions, and their intellectual being … all have to be intact and work in a very

harmonious way." Secondly, Indigenous education needs to provide today's youth with tools to navigate, work, and live within a global world. Educators need to help youth grow their understandings and abilities in meaningful and relevant ways; ideally this will be done with community guidance and involvement ... referred to herein as "knowledge gardening."

THE GUIDING PRINCIPLE OF *ETUAPTMUMK* / TWO-EYED SEEING (E/TES) was long-ago suggested by Elder Albert to help address the above profound educational challenge. He indicates that *Etuaptmumk* in the Mi'kmaw language is the gift of multiple perspectives and he suggests similar understandings probably exist in most Indigenous peoples' languages. As a guiding principle, E/TES encourages the realization that beneficial outcomes are much more likely in any given situation when participants are willing to bring two or more perspectives into play. Albert describes E/TES this way: "I, you, and we ... all need to learn to see from one eye with the best or the strengths in the Indigenous knowledges and ways of knowing ... and to learn to see from the other eye with the best or the strengths in the mainstream (Western or Eurocentric) knowledges and ways of knowing ... but most importantly, we need to learn to see with both these eyes together, for the benefit of everyone (our eco-kin included)."

Work guided by E/TES is not easy and participants require generous time to engage in: (1) co-learning; (2) knowledge scrutinization; (3) knowledge validation; and (4) knowledge gardening. These key essentials are explained as separate categories below but also need to be understood as fluid and wholistic (Figure 1). [Note: holistic is herein spelled with a "w", as wholistic, to emphasize that it means whole, i.e. complete.] Participants should repeatedly ask: "Are we (and how are we) recognizing, acknowledging, and dealing with colonialism, racism, and unequal power dynamics in our discussions, actions, institutions, and communities?" Other challenging questions are indicated for each category.

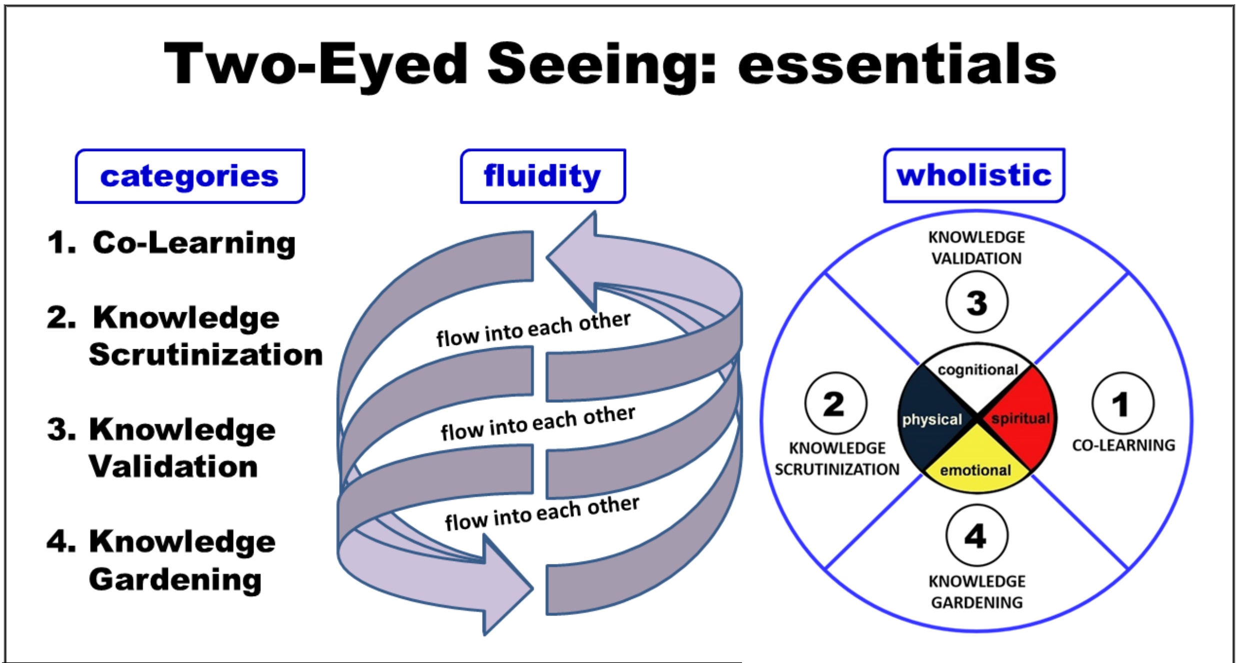

Fig. 1. Key essentials for *Etuaptmumk* / Two-Eyed Seeing

CO-LEARNING is a trans-cultural (and/or inter-cultural, cross-cultural) journey in which the overall intent is to nurture growth of relationships and understandings such that meaningful and respectful collaboration(s) can occur. Participants must be willing to commit to learning together – Indigenous peoples and the newcomers in Indigenous lands – as well as: from each other; knowledge commonalities and differences; how to draw upon the strengths, indeed "the best", in the different ways of knowing, doing, and being; and how to weave back and forth between diverse cultures' actions, values, and knowledges as circumstances require. The journey must be on-going because nurturing genuine relational and collective capabilities requires generous time. Participants further need to develop capacity for *i'l'oqaptmu'k* (from Mi'kmaw) meaning "to revisit to renew, to maintain movement in the direction Spirit intended". These commitments reduce the risk of sliding into a lazy, tokenistic approach in which E/TES and similar efforts become jargon, trivialized, romanticized, co-opted, or used as a "mechanism" (the latter referring to mere assemblage of pieces of knowledge and in a way that lacks the S/spirit of co-learning). Albert encourages that co-learning begin with an exchange of stories, emphasizing such is the foundational basis for all relationships.

Capacity for *I'l'oqaptmu'k* is essential because missteps, differences of opinion, conflicts, and unanticipated challenges are inevitable in any journey. Thus, participants must find ways for the energies of various parties to reach consensus and move forward. In pondering, Albert envisions the example of a Two Bowl Peace Pipe which enables a sacred coming together of the energies of (at least two) participants with the natural energies of the land, water, air, and Spirit. Participants' negative energies will be burned off in moving through the pipe for purification towards consensus. Albert adds insights from Mi'kmaw: *Kisutmajik* – they decided to talk; *kisutmauk* – we come to consensus so we can move forward because we have taken in these natural energies; and *kisutasik* – consensus has been reached. He says *i'l'oqaptmu'k* is truly the essence of co-learning because participants can't work on the basis of assumptions or hearsay. They need to take time to listen to each other rather than merely talking about each other. Deep co-learning and hard work are required ... and must draw upon all four domains of being human: physical, emotional, intellectual, and S/spiritual.

Questions:
- How do we re-awaken S/spirit?
- How do we create opportunities for our S/spirits to start collaborating?
- How do we move to working collectively, rather than as individuals?
- How do we invoke the S/spirit of co-learning in educational settings?
- How do we secure system-wide understandings, encouragement, and support for co-learning?

KNOWLEDGE SCRUTINIZATION is essential if participants are to learn to draw upon the strengths, the best, in their different ways of knowing, doing, and being. Put another way, participants need to become informed, i.e. to know what they are talking about ... it cannot be a chaotic grab bag wherein anything goes. Albert says "our two (or more) paradigms need to be put on the table to be scrutinized. We need honestly to be able to say that the essence, the S/spirit of our ways, has been respected as we work to balance the energies of those ways. We need to put them together." This should involve coming to understand that knowledges are systems and over time move into deeper understandings.

Elder Murdena says visuals are easier for many people to understand than words and encourages that education seek to nurture visual learning skills. Thus, she created a model to emphasize the system nature of Mi'kmaw Knowledge and hers then adapted to serve co-learning and knowledge scrutinization for E/TES (Figure 2). Murdena's MK model has four concentric circles although, she says, traditionally there would be no intentional layering because stories were used to transmit knowledge in a wholistic way. She indicates MK and Western science can share empirical observations of the physical attributes of, for example, a plant and its habitat (outermost circles for both models). In MK, the middle circles of

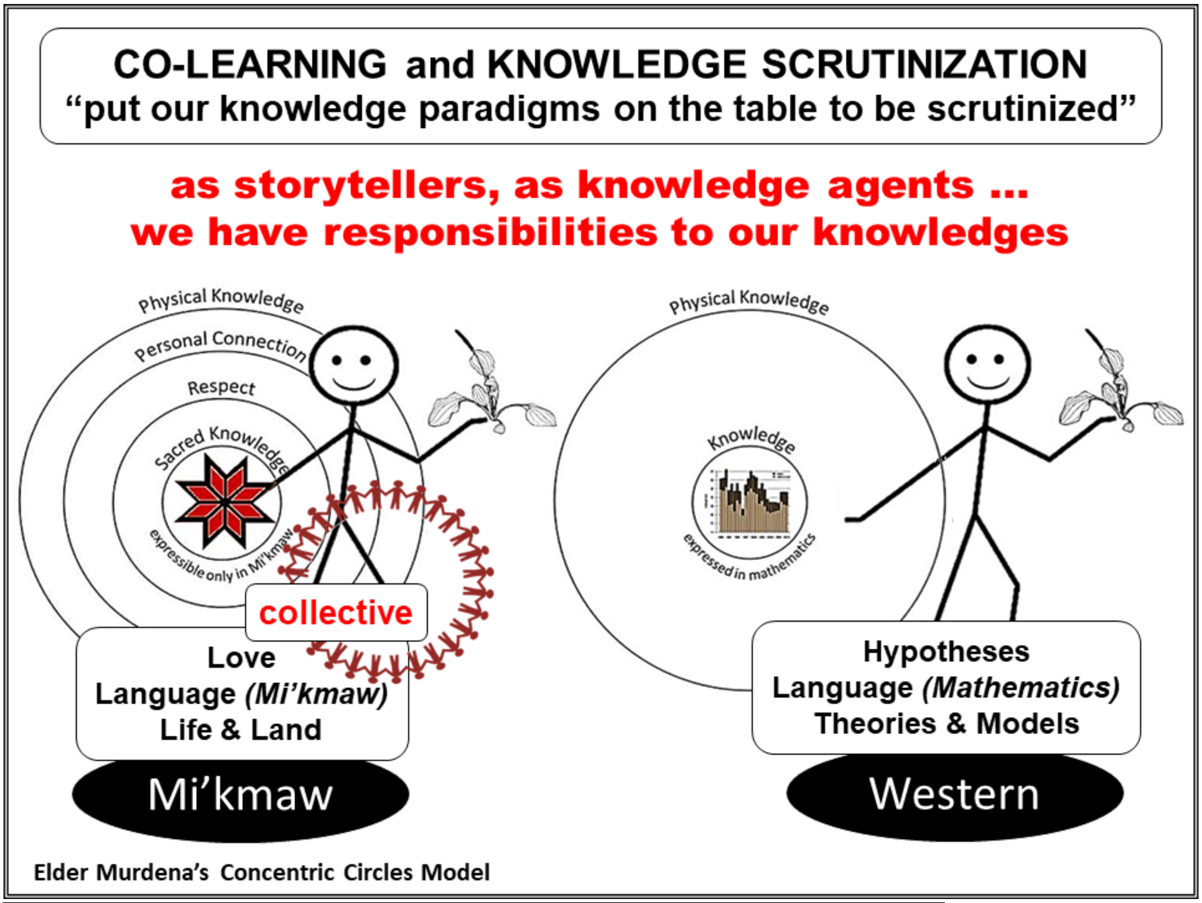

Fig. 2: Models for Mi'kmaw Knowledge and Western Science Knowledge

personal connection and respect are reciprocal plus all four circles are interconnective. Sacred knowledge is innermost, can only truly be understood within the Mi'kmaw language, and generally cannot and should not be translated. Albert says so-called Traditional Ecological Knowledge (TEK) will only ever involve the outer three circles. Western Science uses mathematical language and its model lacks middle circles because subjectivity is maximally diminished. For MK, "the Knowledge Holder / the knower" is an integral participant within the knowledge, thus stands within the model's circles. Albert further emphasizes that MK is collective and thus any one Knowledge Holder has only a small piece. Also, Mi'kmaw people consider their knowledge to be alive and thus both physical and Spiritual, with

the Mi'kmaw language continually reminding Knowledge Holders and users of their responsibilities. In the Western science model, "the knower" stands outside the circles to emphasize objectivity.

Elder Albert, upon reading Shawn Wilson's 2008 book "Research is Ceremony; Indigenous Research Methods" and pondering <u>knowledge systems</u> with Murdena, has brought forward key conceptual understandings within MK, as expressed in the Mi'kmaw language. He suggests all Indigenous languages likely contain understandings akin to these because of the importance of the relationship between the storyteller and the one receiving the knowledge. And thus, Albert suggests: (1) *wsitqamu'kewe'l ankitasuaqnn* for "the nature of thinking or thought; for worldly thoughts or knowledge gained throughout your life" ≈ epistemology; (2) *wsitqamu'kewe'l penawsinn* for "worth of reality; real world; real life" ≈ ontology; (3) *ta'n tel mnsnmen kjiji'taqn,* for "how knowledge is gained" ≈ methodology; and (4) *kjijitaqn ta'n tel wie'wasitew for* "worth of knowledge that will be used" ≈ axiology.

Albert emphasizes that each Indigenous Nation has its own language and thus words which could be more appropriately used in place of "Indigenous Knowledge(s)" or "Traditional Knowledge(s)". For MK, he and Murdena have suggested *Ta'ntelo'lti'k* meaning "the way we *L'nu'k* are."

Knowledge scrutinization requires clarity for key words and concepts. For example, the TK/IS program in seeking to teach post-secondary science by "bringing together Indigenous and Western scientific knowledges and ways of knowing" used this enabling premise: acquisition of scientific knowledge is essential to human survival ... it is a practical engagement with the real world ... and the scientific pursuit of knowledge must, therefore, be as old as the consciousness of our human species. The program worked within the broadened view of science as "dynamic, pattern-based knowledge shared through stories about our interactions with and within nature" for the Indigenous and Western sciences. Curricula evolved with explorations of common ground (outermost circles in the knowledge models) along with acknowledgement and respect for differences (remaining circles). Discussion included roles and responsibilities of knowledge agents as storytellers and a conceptual framework was developed featuring pattern recognition, transformation, and expression. Resources and sources were diverse: the Mi'kmaw language, Indigenous Elders and Western scientists as guest speakers, community workshops, literature, the land, and more.

Questions:
- How can we guard against negativism in this scrutiny?
- How can we encourage critics and skeptics to engage, and thus come to understand rather than fear or reject?

<u>KNOWLEDGE VALIDATION</u> is essential within all knowledge systems and Indigenous Elders and Knowledge Holders are as interested in knowledge integrity as are mainstream academics and researchers; validation by peer-review is the only acceptable means. The processes in mainstream academia are well understood albeit some aspects always engender debate and controversy. Peer-review within Indigenous Knowledges uses processes specific to a Nation, region, or community and tend to be unknown or poorly understood within mainstream academia. In Atlantic Canada, Elders from

various Nations worked together in 2009-2011 to provide formal recommendations about Indigenous Knowledges; Elders were clear: they wanted to be consulted or actively involved whenever their knowledge(s) was(were) being discussed or used. Their work has become known as the "Elders Eight Recommendations for Honouring Traditional Knowledge" and their recommendations were supported by the Atlantic Chiefs (see APCFNC website). Elder Albert further indicates: Elders and Knowledge Holders will never do anything to compromise the integrity of their knowledge because it is their culture, language, youth, and communities at stake. He insists that genuine Elders and Knowledge Holders will always act to ensure accuracy, authenticity, and sacredness.

Questions:

- What assistance might Indigenous Elders and Knowledge Holders want to implement the knowledge validation they insist upon?
- How does validation fit within the wholism of oral knowledge transmission?

KNOWLEDGE GARDENING is the co-learning-informed, action-based, participatory-work of growing E/TES understandings within meaningful projects relevant to or grounded in communities. Ideally, therefore, knowledge gardening is community-oriented, -based, and/or -directed. Education provides perfect grounds. "Gardening" is the word of choice because it denotes an overall organic context with emergence of properties and products, some anticipated and some not, some desirable and some otherwise ... i.e., a genuinely dynamic learning environment. A garden, moreover, is generally understood within a vision for positive outcomes. The vision, to advance, will require a nurturing environment, appropriate time, and participatory gardeners. A garden is also situation-specific plus interrelationship-dependent, taking on dramatically different shape depending upon participants (both humans and plants), circumstances, resources, and much more. Gardening is an activity that naturally draws upon all four aspects of being human ... resonating with Albert's earlier words that "to have a whole person, their Spirit, their physical being, their emotions, and their intellectual being … all have to be intact and work in a very harmonious way." And, the phrase "knowledge gardening" is richly congruent with Albert's statement that Mi'kmaw Knowledge is alive and thus both Spiritual and physical. It's equally congruent with Murdena emphasizing that Mi'kmaw Knowledge grows and changes through time. Finally, gardening leads to tangible and intangible products … an understanding that resonates with Albert indicating "knowledge lives in stories" and the phrase "story knowledge".

Gardens generally have pathways. Within the TK/IS educational program and its affiliated research, there were many pathways and much story knowledge emerged (Bartlett et al., 2012, 2015; IS website; Hatcher et al., 2009; Iwama et al., 2009; Marshall et al., 2010; 2015). Another educational example is with Mi'kmaw Kina'matnewey (the Mi'kmaw educational authority in NS) and its work to create E/TES science curricula with community schools. Currently in the early stages, its story knowledge will grow over time. Additional NS examples, albeit potential only (as they do not self-identify as "knowledge gardening") are: (1) *Netukulimk* within moose management and other programs of the Unama'ki Institute of Natural Resources; (2) the Collaborative Environmental Planning Initiative for the Bras d'Or Lake and watershed; and (3) interpretive planning for the Mi'kmawey Debert Cultural Centre. These three are outside formal educational settings (internet searches will reveal information about each).

Both formal and informal learning are key in Indigenous wholistic lifelong learning as portrayed in the First Nations Holistic Lifelong Learning Model of the Aboriginal Learning Knowledge Centre of the Canadian Council on Learning (see CCL-AbLKC reference). Two additional models, one each for Métis and Inuit understandings, were also created.

Questions:

- How can we create opportunities to grow, together ... and help each other?
- How can we re-awaken understandings that healing comes from within?
- How can we harness the power of story?
- How can we learn to "listen to understand" rather than simply listening to say something back?
- How can we engage our responsibility to reflect?
- How can we learn that truly, we are our stories, that everything is story?
- How can we learn to listen to stories ... to share stories ... to co-learn from stories?

OVERALL CONCLUDING WORDS come from Elder Albert: "The work of E/TES is not easy. And so we need to understand that sometimes our most important job is to plant seeds for the future, for the youth, knowing seeds germinate when the time is right. Our work must, therefore, be ongoing. *Msɨt No'kmaq.*"

REFERENCES

APCFNC: http://www.apcfnc.ca/images/uploads/ResearchSummary-HonouringTraditionalKnowledgeResearchSummary.pdf

Bartlett, C., Marshall, M., Marshall, A., and Iwama, M. 2015. Integrative Science and Two-Eyed Seeing: Enriching the Discussion Framework for Healthy Communities. Chapter 10 (pp 280-326) in "Ecosystems, Society and Health: Pathways through Diversity, Convergence and Integration", edited by Lars K. Hallstrom, Nicholas P. Guehlstorf, and Margot W. Parkes, McGill-Queen's University Press, Montreal & Kingston.

Bartlett, C., Marshall, M., and Marshall, A. 2012. Two-Eyed Seeing and other Lessons Learned within a co-learning journey of bringing together indigenous and mainstream knowledges and ways of knowing. Journal of Environmental Studies and Sciences, 2(4): 331-340.

CCL-AbLKC: http://www.afn.ca/uploads/files/events/fact_sheet-ccoe-4.pdf

Hatcher, A., Bartlett, C.M., Marshall, A., and Marshall, M. 2009. Two-Eyed Seeing in the classroom environment: concepts, approach and challenges. Canadian Journal of Science, Mathematics, and Technology Education, 9(3): 141-153.

IS: http://www.integrativescience.ca

Iwama, M., Marshall, A., Marshall, M., and Bartlett, C. 2009. Two-Eyed Seeing and the Language of Healing in Community-Based Research. Canadian Journal of Native Education, 32: 3-23.

Marshall, M., Marshall, A., and Bartlett, C. 2015. Two-Eyed Seeing in Medicine. Chapter 2 (pp 16-24) in "Determinants of Indigenous Peoples' Health in Canada; beyond the social", edited by Margo Greenwood, Sarah de Leeuw, Nicole Marie Lindsay, and Charlotte Reading. Canadian Scholars Press, Toronto.

Marshall, A., Marshall, M., and Iwama, M. 2010. Approaching Mi'kmaq Teachings on the Connectiveness of Humans and Nature. In: S. Bondrup-Nielsen, K. Beazley, G. Bissix, D. Colville, S. Flemming, T. Herman, M. McPherson, S. Mockford, and S. O'Grady (Eds). 2010. Ecosystem Based Management: Beyond Boundaries. Proceedings of the Sixth International Conference of Science and the Management of Protected Areas, 21–26 May 2007, Acadia University, Science and Management of Protected Areas Association, Wolfville, NS.
