# Supplementary material for: How is Etuaptmumk/Two-Eyed Seeing characterized in Indigenous health research? A scoping review
Source: PLoS One. 2021 Jul 20;16(7):e0254612. doi: 10.1371/journal.pone.0254612 (PMC8291645; doi:10.1371/journal.pone.0254612)
Supplement: S2 File — List of Elder Albert Marshall and Elder Murdena Marshall’s presentations, panels, and publications about Two-Eyed Seeing. (DOC) [file pone.0254612.s002.doc]

**S2 File. List of Elder Albert Marshall and Elder Murdena Marshall’s presentations, panels, and publications about Two-Eyed Seeing**

**2019**

Bartlett, C., and Marshall, A. 2019. Etuaptmumk / Two-Eyed Seeing. Building Capacity for Reconciliation: transforming teaching and learning through Etuaptmumk. Workshop organized by FoodARC (MSVU) and Atlantic Indigenous Mentorship Network, Mount Saint Vincent University, Halifax, NS. 9 January 2019 [Invited Workshop Keynote, plus Daylong Presentation]

Marshall, A. and Bartlett, C. 2019. Etuaptmumk / Two-Eyed Seeing … transforming teaching and learning. Mount Saint Vincent University President’s Visiting Lecture Series on Teaching and Learning in partnership with FoodARC (MSVU) and Atlantic Indigenous Mentorship Network. Mount Saint Vincent University, Halifax, NS. 9 January 2019. [Invited Public Lecture]

**2018**

Bartlett, C., and Marshall, A. 2018. Planting Seeds – Reconciliation in Canada today: considerations on Etuaptmumk / Two-Eyed Seeing and Integrative Science. Indigenous Knowledge and Access Symposium, co-hosted by Dalhousie Libraries, Dalhousie's School of Information Management, and Library and Archives Canada. Halifax, NS. 15 November 2018.

[Invited Keynote]

Marshall, A. 2018. *Etuaptmumk* / Two-Eyed Seeing. Special Presentation for DFO-CCG Labour Relations Symposium, Halifax, NS. 30 May 2018. [Invited Informant]

Bartlett, C. and Marshall, A. 2018. Storytelling and Dancing: valuing our relationships and actions → Co-Learning. Association of Nova Scotia University Teachers Association, Membertou, NS, 3-4 May 2018. [Invited Presentation and Panel Participant]

Bartlett, C. and Marshall, A. 2018. Two-Eyed Seeing. Policy Community Conference, Government of Canada, Ottawa, ON. 28-29 March 2018. [Invited Panel Participants]

Marshall, A. 2018. Learning Together by Learning to Listen to Each Other (in English, Mi’kmaw, and French). “Network Voices” overall Introduction to special issue on Truth and Reconciliation. *Education Canada Magazine* 58 (2): 6-7. [Invited Article]

Marshall, A. 2018. Nurturing transformative action through sharing understandings. The Compassionate Community or “Living as if We give a Damn” series, Cape Breton University, Sydney, NS. 16 March 2018. [Invited Presenter]

Marshall, A. 2018. *Etuaptmumk* / Two-Eyed Seeing. Gwen Bear Memorial Lecture, University of New Brunswick, Fredericton, NC. 18 January 2018. [Invited Speaker]

Marshall, A. 2018. *Etuaptmumk* / Two-Eyed Seeing. Class lecture for Professor Dave Perley, University of New Brunswick, Fredericton, NC. 17 January 2018. [Invited Lecturer]

Marshall, A. 2018. *Etuaptmumk* / Two-Eyed Seeing. President’s Lecture, Mount Allison University, Sackville, NB. 16 January 2018. [Invited Speaker]

Marshall, A. and Bartlett, C. 2018. Storytelling and Dancing: valuing our relationships and actions → Co-Learning. Association of Nova Scotia University Teachers Association, Membertou, NS, 3-4 May 2018. [Invited Presenter]

Marshall, A. and Bartlett, C. 2018. *Etuaptmumk* / Two-Eyed Seeing for Knowledge Gardening. Encyclopedia of Educational Philosophy and Theory (Springer online), edited by Michael A. Peters within section “Indigenous Education in Canada” coordinated by Michelle Hogue. [Invited Article]

Marshall, A., Knockwood, C., and Bartlett, C. 2018. The Best of Both Worlds; bringing Mi’kmaw Knowledge and Western curriculum together. Article in special issue on Truth and Reconciliation. *Education Canada Magazine* 58 (2): 26-29. [Invited Article]

**2017**

Marshall, A. 2017. Two-Eyed Seeing – a guiding principle for inter-cultural collaboration. Climate change, drawdown and the human prospect: a retreat for empowering our climate future for rural communities; sponsored by the Thinkers Lodge. Pugwash, NS. 28 September – 1 October 2017. [Invited Elder and Key Speaker]

Marshall, A. 2017. Two-Eyed Seeing and Co-Learning. “Indigenous Journey: Healing from Trauma” Workshop, held in partnership by The First People’s Wellness Circle and Thunderbird Partnership Foundation. Vancouver, BC. 29 May – 2 June 2017. [Invited Elder and Speaker]

Marshall, A. 2017. Two-Eyed Seeing and Co-Learning. Newfoundland and Labrador Environmental Educators, Annual Workshops and AGM, 2017 theme: Integrating Traditional Knowledge in the Classroom, St. John’s, NL. 25-27 May 2017. [Invited Elder and Keynote Speaker]

Marshall, A. 2017. Two-Eyed Seeing and Co-Learning. *L’nui’sultinej* (Let Us Speak Mi’kmaq) Conference, Eskasoni First Nation, NS. 17-19 May 2017. [Invited Elder and Speaker]

Marshall, A. 2017. Two-Eyed Seeing and Co-Learning. Halifax Regional School Board - Culturally Relevant Pedagogy Symposium: Improving Student Achievement Through Critical Consciousness, Collaborative Action and Community Engagement. Halifax, NS. 11-12 May 2017. [Invited Elder and Speaker]

Marshall, A. 2017. Two-Eyed Seeing and Co-Learning. Two Eyed Seeing. Discussion on Clearcutting in Nova Scotia, Inverness, NS. 4 April 2017. [Invited Panel Speaker]

Marshall, A. 2017. Truth and Reconciliation: Sharing Understandings. Special College and Community Event, Nova Scotia Community College, Port Hawkesbury, NS. 28 March 2017. [Invited Elder and Speaker]

Marshall, A. 2017. Two-Eyed Seeing and Co-Learning. Classrooms discussions. Trent University, Peterborough, ON. 13-16 March 2017. [Visiting Guest Elder]

Marshall, A. 2017. Considering an Elders’ Senate for the “Atlantic Indigenous Mentorship Network” CIHR proposal by Dr. Debbie Martin, Dalhousie University. Elders’ Gathering, Millbrook First Nation, NS. 9 March 2017. [Invited Key Elder]

Marshall, A. 2017. Two-Eyed Seeing in Medicine. Interview by the National Collaborating Centre for Aboriginal Health of chapter author in book "Determinants of Indigenous Peoples’ Health in Canada; beyond the social", edited by Margo Greenwood, Sarah de Leeuw, Nicole Marie Lindsay, and Charlotte Reading. Canadian Scholars Press, Toronto. <https://vimeo.com/196661196> [On-line Interview, posted February 2017]

Marshall, A. 2017. Truth and Reconciliation: Sharing Understandings for St. Francis Xavier University. Special Event, Antigonish, NS. 20 January 2017. [Invited Elder and Panel Speaker]

**2016**

Marshall, A. 2016. Elder’s Voice at CEPI’s (Collaborative Environmental Planning Initiative’s) “Sustainability Practices Conference” entitled “The People of the Lakes Speak”, Wagmatcook First Nation, NS, and Baddeck, NS. 8-10 November 2016. [Invited CEPI Elder]

Marshall, A. 2016. What If Evaluation Could Start Over. Panel on “Evaluation on the Edge” for Canadian Evaluation Society, Newfoundland and Labrador Chapter, St. John’s, NL. 5-8 June 2016. [Invited Elder and Panel Participant]

Marshall, A. and Bartlett, C. 2016. Two-Eyed Seeing: Essentials and Challenges. CIHR Proposal Development Meeting of Dr. Debbie Martin, Dalhousie University for proposal entitled “Atlantic Indigenous Mentorship Network”, Halifax, NS. 28 September 2016. [Invited Key Presenters]

Marshall, A. and Bartlett, C. 2016. Two-Eyed Seeing: Essentials and Challenges. CIHR Proposal Development Meeting of Dr. Debbie Martin, Dalhousie University for proposal entitled “Atlantic Indigenous Mentorship Network”, Membertou First Nation, NS. 7 October 2016. [Invited Key Presenters]

Marshall, A. and Bartlett, C. 2016. Two-Eyed Seeing: Essentials and Challenges. CIHR Proposal Development Meeting of Dr. Debbie Martin, Dalhousie University for proposal entitled “Atlantic Indigenous Mentorship Network”, Happy Valley – Goose Bay, NL. 18 October 2016. [Invited Key Presenters]

**2015**

Bartlett, C., Marshall, M., Marshall, A., and Iwama, M. 2015. Integrative Science and Two-Eyed Seeing: Enriching the Discussion Framework for Healthy Communities. Chapter 10 (pp. 280-326) in "Ecosystems, Society and Health: Pathways through Diversity, Convergence and Integration". Edited by Lars K. Hallstrom, Nicholas Guehlstorf, and Margot Parkes, McGill-Queen’s University Press. [Invited Book Chapter, peer-reviewed]

Marshall, A. 2015. Mi’kmaq Language, Culture, and Healing – Integrative Science – Co-Learning – *Etuaptmumk* or Two-Eyed Seeing. First Peoples’ House of Learning and the Department of Indigenous Studies, Trent University, Peterborough, ON. 1 December 2015. [Invited Elder and Speaker]

Marshall, A. 2015. Mi’kmaq Language, Culture, and Healing – Integrative Science – Co-Learning – *Etuaptmumk* or Two-Eyed Seeing. Four Directions Aboriginal Students’ Centre and the Health, Environment, and Communities Research Lab, Queen’s University, Kingston, ON. 1 December 2015. [Invited Elder and Speaker]

Marshall, A. 2015. Mi’kmaq Language, Culture, and Healing – Integrative Science – Co-Learning – *Etuaptmumk* or Two-Eyed Seeing. First Nations’ House, University of Toronto, Toronto, ON. 30 November 2015. [Invited Elder and Speaker]

Marshall, A.2015. Two-Eyed Seeing. Seminar for School of the Environment, Saint Mary’s University, Halifax, NS. 28 January 2015. [Invited Presenter]

Marshall, M., Marshall, A., and Bartlett, C. 2015. Two-Eyed Seeing in Medicine. Chapter 2 (pp 16-24) in "Determinants of Indigenous Peoples’ Health in Canada; beyond the social", edited by Margo Greenwood, Sarah de Leeuw, Nicole Marie Lindsay, and Charlotte Reading. Canadian Scholars Press, Toronto. [Invited Book Chapter, peer-reviewed]

**2014**

Bartlett, C.M., Marshall, A., and Marshall, M. 2014. *Etuaptmumk* - Two-Eyed Seeing: where Indigenous and Western perspectives meet. "Welcome to our Talking Circle" speaker series of the Mi'kmaq-Maliseet Institute at the University of New Brunswick, Fredericton, NB. 16 April 2014. [Invited Guest Speakers]

Marshall, A. and Marshall, M. 2014. Two-Eyed Seeing. Mi’kmaw Kina’matnewey Strategic Planning Symposium for “Elders In The Classroom”, Dartmouth, NS. 5-7 March 2014. [Key Invited Elders]

Marshall, A. 2014. Mi'kmaq Traditional Knowledge and Two-Eyed Seeing as a guiding principle to begin to incorporate ATK (Aboriginal Traditional Knowledge) into DFO's planning process for the commercial fisheries in Atlantic Canada. Gespe'gewaq Mi'gmaq Resource Council (GMRC) Science Symposium, Miramichi, NB. 25 February 2014 [Invited Elder and Speaker]

**2013**

Bartlett, C., Marshall, A., and Marshall, M. 2013. Bringing ATK into DFO’s Fisheries Management Planning Cycle for Snow Crab (Gulf Management Area 12). Atlantic Policy Congress of First Nations Chiefs (APCFNC) Fisheries Workshop, Moncton, NB. 5-6 February 2013. [Invited Workshop Presentation]

Bartlett, C., Marshall, A., and Marshall, M. 2013. Roadmap for Incorporating Aboriginal Traditional Knowledge (ATK) into DFO's "Integrated Fisheries Management Plan" planning process for the Commercial Snow Crab Fishery in the Southern Gulf of St. Lawrence (Area 12). Report for Atlantic Policy Congress of First Nations Chiefs (APCFNC). 20 March 2013.

[Final Report for Commissioned Consultancy]

Marshall, A. 2013. Did eels change the course of history? Elder Albert speaks. **Wagmatcook Lecture Series, Wagmatcook First Nation, NS. 28 November 28** 2013. [Invited Elder Speaker at launch of UINR / Parks Canada video]

Marshall, A., and Kavanagh, S. 2013. Two Eyed Seeing:  *Netukulimk* and Stewardship.  Workshop on Species at Risk Stewardship in Atlantic Canada:  Best Practices, Collaborative Approaches, and Techniques that Work, hosted by the Mersey Tobeatic Research Institute. Wolfville, NS.  15-16 May 2013. [Invited Workshop Presentation]

Marshall, A. 2013. Mi’kmaq Traditional Knowledge. 2nd National Colloquium on Indigenous Knowledge and Aboriginal Health Research, sponsored by AHRNetS (Aboriginal Health Research Network Secretariat) for Canadian Institutes of Health Research (CIHR)-funded NEAHRs (Network Environments for Aboriginal Health Research), co-sponsored by territorial hosts Kloshe Tillicum BC NEAHR and Alberta NEAHR, Vancouver, BC. 12-13 March 2013.

[Invited Colloquium Speaker]

Marshall, A. 2013. Mi’kmaq Traditional Knowledge and Two-Eyed Seeing. Training session for commercial fishers re "commercial fishing and species at risk", provided by UINR (Unama'ki Institute of Natural Resources) and Crane Cove Seafoods, in conjunction with presentations by Canadian Sea Turtle Network, Marine Animal Response Society, and the World Wildlife Fund. Eskasoni First Nation, NS. 27 February 2013. [Invited Training Session Presentation]

Marshall, A., and Lefort, N. 2013. Two-Eyed Seeing: Putting Multiple Perspectives into Practice EECOM (Environmental Education and Communication) Conference, with theme "Environmental Learning and Connections among nature(s), culture(s), and well-being(s)", University of Victoria, Victoria, BC. 26-30 June 2013. [Refereed Conference Presentation]

Marshall, M., and Marshall, A. 2013. Two-Eyed Seeing, *Netukulimk*, and Mi’kmaq Traditional Knowledge. Guest Elders in Residence, Trent University, Peterborough, ON. 14-17 October 2013. [Invited Elders and Guest Speakers – several university courses plus public events]

**2012**

Bartlett, C., Marshall, M., and Marshall, A. 2012. Two-Eyed Seeing and other Lessons Learned within a co-learning journey of bringing together indigenous and mainstream knowledges and ways of knowing. Journal of Environmental Studies and Sciences, 2(4): 331-340. [Peer-reviewed Article]

**Bartlett, C., Marshall, M., and Marshall, A. 2012.** Co-Learning and Two-Eyed Seeing for Aboriginal Health Research. Presentation within research team meeting "Our Ancestors Are In Our Water, Land, and Air: a Two-Eyed Seeing Approach to Researching Environmental Health Concerns with Pictou Landing First Nation" (CIHR-funded research project of Dr. Heather Castleden), Tatamagouche, NS. 28-29 April 2012. [Invited Workshop Presentation]

Bartlett, C., Marshall, M., and Marshall, A. 2012. Moving forward with Elders' Recommendations from APCFNC Elders Research Project "Honouring Traditional Knowledge" - considerations from Two-Eyed Seeing and Co-Learning for "Honouring Traditional Knowledge in Academia". Presentation for AAEDIRP’s (Atlantic Aboriginal Economic Development Integrated Research Program's) university partners and others; organized by AAEDIRP and APCFNC (Atlantic Policy Congress of First Nations Chiefs) Secretariat, Cole Harbour, NS, 30 March 2012. [Invited Oral Presentation]

Marshall, A. 2012. Etuaptmumk / Two-Eyed Seeing. Public presentation for Environmental Science Program. Saint Mary's University, Halifax, NS, 15 Nov 2012. [Invited Public Presentation]

Marshall, A. 2012. Two-Eyed Seeing and Co-Learning.  Presentation for course "Introduction to Aboriginal Peoples' Health and Healing" (Inter-Professional Health Education 2201), Faculty of Health.  Dalhousie University, Halifax, NS, 29 October 2012. [Invited Oral Presentation]

Marshall, A. 2012. Two-Eyed Seeing and Co-Learning.  Presentation within Aboriginal Health Research Summer Institute for theme "Walking Our Path:  Indigenous and Decolonizing Methodologies" organized by Atlantic Aboriginal Health Research Program (AAHRP) . Pictou Lodge, Pictou, NS, 28 May - 1 June 2012.  [Invited Keynote Speaker]

Marshall, A. 2012. *Etuaptmumk* / Two-Eyed Seeing. Special presentation within permaculture course at Blockhouse, NS; sponsored by The Blockhouse School - a project of the South Shore Social Ventures Co-op, 21 May 2012. [Invited Oral Presentation]

Marshall, A. 2012. *Etuaptmumk* / Two-Eyed Seeing. Presentation at Earth Day Celebrations. Kluscap Heritage Centre, Millbrook First Nation, NS.  20 April 2012. [Invited Keynote Speaker]

Marshall, A. 2012. Two-Eyed Seeing / *Etuaptmumk:* Mi'kmaw Traditional Knowledge for Today. Lands Management and Economic Development Conference; Atlantic Region Aboriginal Lands Association. Moncton, NB, 7 March 2012. [Invited Speaker]

Marshall, M., and Marshall, A. 2012. Two-Eyed Seeing / *Etuaptmumk:* Mi'kmaw Traditional Knowledge. Presentations for junior high school students and discussions with Elders in Chapleau Cree First Nation, ON, 13-17 November 2012. [Invited Elders and Speakers]

Marshall, M., and Marshall, A. 2012. Two-Eyed Seeing / *Etuaptmumk:* Mi'kmaw Traditional Knowledge. Presentation and meeting discussions for planning re Chapleau Crown Game Preserve in Chapleau Cree First Nation, ON, 13-17 February 2012. [Invited Elders and Guest Speakers]

Marshall, M., Marshall, A., and Bartlett, C. 2012. *Etuaptmumk* / Two-Eyed Seeing ... and Mi'kmaw Traditional Knowledge. Response to special presentation by Don Fiddler re "working on the determinants of Aboriginal health and education: building resilient youth and families", a Frank McKenna Centre for Leadership Encounter sponsored by the National Collaborating Centre for Determinants of Health in conjunction with the Canada Research Chair in Indigenous Peoples and Sustainable Communities at StFXU. St. Francis Xavier University, Antigonish, NS, 19 November 2012. [Invited Responders]

**2011**

Austin, G., Marshall, A., Marshall, M. and other Elders. 2011. “Honouring Traditional Knowledge” - Elders’ Recommendations emergent from research project of APCFNC (through AAEDIRP, Atlantic Aboriginal Economic Development Integrated Research Program). Atlantic First Nations’ Annual Health Conference - Walking the Path to Wellness. Dartmouth, NS, 14-16 November 2011. Recommendations and project description available at: [http://www.apcfnc.ca/en/resources/HonouringTraditionalKnowledgeFinal.pdf](https://owa.cbu.ca/exchweb/bin/redir.asp?URL=http://www.apcfnc.ca/en/resources/HonouringTraditionalKnowledgeFinal.pdf)

[Invited Oral Presentation]

**Bartlett, C., Marshall, M., and Marshall, A. 2011.** Healing and Two-Eyed Seeing. Class presentation for Dr. Jim Gerrie - Religious Studies 267 / Philosophy 267 - BSc Nursing students. Cape Breton University, Sydney, NS, 17 February 2011. [Invited Oral Presentation]

Marshall, A. 2011. Two-Eyed Seeing. Aboriginal Forum – Insight (strategic planning, economic development, community sustainability, water management, etc). Halifax, NS, 5-6 December 2011. [Invited Keynote Luncheon Address]

Marshall, A. 2011. Mobilizing Pathways to Reconciliation: Knowledge Circle 5 - Knowledge and Rights. Truth and Reconciliation Commission of Canada - National Atlantic Event, Halifax, NS, 26-29 October 2011. [Invited Key Elder and Speaker]

Marshall, A. 2011. Healthy Land, Healthy People: Forging International Connections - gathering organized by the National Collaborating Centre for Aboriginal Health (NCCAH). University of British Columbia, Vancouver, BC. 3-5 October 2011. [Invited Participant] [http://nccah.netedit.info/264/Healthy_Land__Healthy_People_-_International_Gathering.nccah](http://nccah.netedit.info/264/Healthy_Land__Healthy_People_-%09_International_Gathering.nccah)

Marshall, A. 2011. Two-Eyed Seeing. 10^th^ International Conference on Mercury as a Global Pollutant. Halifax, NS, 24-29 July 2011. [Invited Speaker]

Marshall, A. 2011. Two-Eyed Seeing - *Etuaptmumk*. "Mainstreaming Indigenous Knowledge for Sustainability" conference to discuss taking a Red Path toward a Green Society. The Johnson Foundation at Wingspread, Racine, WI, USA, 9-11 May 2011. [Invited Participant and Speaker]

Marshall, A. 2011. Mi’kmaw traditional understandings with respect to bio-mass. Meeting on biomass with Nova Scotia First Nations’ Chiefs organized by KMK (Kwilmu'kw Maw-klusuaqn - Mi'kmaq Rights Initiative) and the Ecology Action Centre (Halifax). Truro, NS. 6 April 2011. [Invited Participant and Speaker; postponed due to scheduling conflicts]

Marshall, A. 2011. Mi’kmaw traditional understandings with respect to human rights. Truro, NS, 28 March 2011. [Invited Speaker]

Marshall, A. 2011, Two-Eyed Seeing. Canadian Aboriginal AIDS Network Wise Practices III HIV/AIDS Research Conference; Creativity + Research = Positive Action Wise Practices III. Halifax, NS, 9-11 March 2011. [Invited Speaker]

**Marshall, A. 2011. National Round Table on the Environment and the Economy (NRTEE). Workshop hosted by** NRTEE in collaboration with the Bras d’Or Lakes Collaborative Environmental Planning Initiative (CEPI). Membertou First Nation, NS, 2 March 2011. [Invited Workshop Participant]

Marshall, A. and Bartlett, C. 2011. Two-Eyed Seeing (an old-new way of bringing together different perspectives) and Integrative Science. Environment Canada – Aboriginal Traditional Knowledge learning sessions. Dartmouth, NS, 1 December 2011. [Invited Oral Presentation]

Marshall, A., and Bartlett, C. 2011. Sharing Our Stories: Two-Eyed Seeing and Co-Learning to bring together Indigenous Traditional Knowledges and Western Science. Mi'kmaw Cultural Lecture Series, Wagmatcook First Nation, NS, 28 April 2011. [Invited Public Presentation]

**Marshall, A., and Bartlett, C. 2011.** Traditional Ecological Knowledge, Two-Eyed Seeing, Co-Learning. National Aboriginal Fisheries Forum - opportunities, challenges, solutions. Hosted by Atlantic Policy Congress of First Nations Chiefs Secretariat with Assembly of First Nations, Aboriginal Aquaculture Association, BC First Nations Fisheries Council, and Native Brotherhood of BC. Dartmouth, NS, 29-31 March 2011. [Invited Special Presentation]

**Marshall, A., and Bartlett, C. 2011.**  A Co-Learning Journey: Two-Eyed Seeing and Integrative Science. Mawi Wiqsonultine * Mamu Uauitshitutua * Mawi Apo'qnmatultinej * Llonnataikajuttigelautta (Let's All Help Each Other): a conference on Aboriginal community-based research. Atlantic Aboriginal Economic Development Integrated Research Program (AAEDIRP) and Atlantic Policy Congress of First Nations Chiefs Secretariat; Moncton, NB, 16-18 March 2011. [Invited Conference Keynote]

**Marshall, A., and Bartlett, C. 2011.** Two-Eyed Seeing and Co-Learning. DFO (Maritime) Aboriginal Fisheries Guardian Training. Canadian Coast Guard College, Sydney, NS, 1 March 2011. [Invited Oral Presentation]

Marshall, A., and Marshall, M. 2011. Mi’kmaw medicinal plants. Presentation for NewPage Port Hawkesbury Corp. (NewPage), other participants in the forest industry, and the general public. Sydney, NS, 31 March 2011. [Invited Presentation]

Marshall, M. 2011. L'nuita'si: Mi'kmaw Tribal Consciousness. In "Ta'n Wetapeksi'k: Understanding From Where We Come", Proceedings of the 2005 Debert Research Workshop, Debert, Nova Scotia, Canada (Chapter 16; pp. 173-177); edited by T. Bernard, L.M. Rosenmeier, and S.L. Farrell. Eastern Woodland Print Communications, Truro, NS. [Book Chapter] (based on 2005 workshop presentation)

**2010**

Bartlett, C., and Marshall, A. 2010. Two-Eyed Seeing Science Curricula. Community Education Strategic / Operational Planning Symposium for “Language and Culture” of Mi’kmaw Kina’matnewey. Dartmouth, NS, 11 March 2010. [Invited Oral Presentation]

Bartlett, C., and Marshall, A. 2010. Integrative Science and Two-Eyed Seeing: Walking & Talking Together. Forum 2 for CFR (Community Fisheries Representative) program and AFSAR (Aboriginal Funds for Species at Risk) organized by UFFCA (Upper Fraser Fisheries Conservation Alliance). Prince George, BC, 4 March 2010. [Invited Oral Presentation]

Bartlett, C., and Marshall, A. 2010. Integrative Science and Two-Eyed Seeing: Walking & Talking Together. Cultural Awareness Training Session for ATK (Aboriginal Traditional Knowledge) for Fisheries & Oceans Canada – SARA (Species At Risk Act) Working Group. Environment Canada, Vancouver, BC, 2 March 2010. [Invited Oral Presentation Invited]

Bartlett, C., and Marshall, A. 2010. Two-Eyed Seeing: “taking down the boundaries” between Mi’kmaq Traditional Knowledge and the mainstream. AAEDIRP (Atlantic Aboriginal Economic Development Integrated Research Program) Let’s Work Together: A Conference on Creating Meaningful and Sustainable Employment for Atlantic Aboriginal People. Dartmouth, NS, 16-18 February 2010. WikiProceedings at: [http://aaedirpletsworktogetherconference.wikispaces.com](http://aaedirpletsworktogetherconference.wikispaces.com/) [Invited Oral Presentation]

Bartlett, C., and Marshall, A. 2010. Traditional Knowledge meets Western Knowledge: Two-Eyed Seeing – an old-new way of bringing together different perspectives. Learning Seminar on “Mi’kmaq Ecological Knowledge: How to Include this Valuable Resource in Your Work” organized by Government of Nova Scotia – Office of Aboriginal Affairs. Halifax, NS, 4 February 2010. [Invited Oral Presentation]

Bartlett, C., and Marshall, A. 2010. Making Our Way: Patterns of Interconnectiveness. YouthLink Conference on “Accessing Opportunities to Post-Secondary Education” organized by MMAYC (Mi’kmaq Maliseet Atlantic Youth Council) and Atlantic Policy Congress of First Nation Chiefs Secretariat. Moncton, NB, 22-24 January 2010. [Invited Oral Presentation]

Bartlett, C., Marshall, M., and Marshall, A. 2010. Spirits of Health. “Taking the Next Steps: Sustainability Planning, Policy and Participation for Rural Canadian Communities” organized by the Alberta Centre for Sustainable Rural Communities of the University of Alberta. Augustana Campus – University of Alberta, Camrose, AB, 21-23 October 2010. [Oral Presentation]

Harris, P., Bartlett, C., Marshall, M., and Marshall, A. 2010. Mi’kmaq Night Sky Stories; patterns of interconnectiveness, vitality and nourishment. Communicating Astronomy to the Public Journal (CAPjournal), no. 9 (October), 14-17. [Article]

Marshall, A. 2010. Two-Eyed Seeing (an old-new way of bringing together different perspectives) and Integrative Science (Part 1). Environment Canada Aboriginal Peoples and the Environment Speakers Series – Aboriginal Traditional Knowledge (event held in partnership with the Department of Fisheries and Oceans). Gatineau, QC, 7 December 2010. [Invited Oral Presentation]

Marshall, A. 2010. Two-Eyed Seeing. Sunday Service, Universalist Unitarian Church of Halifax. Halifax, NS, 5 December 2010. [Invited Oral Presentation]

Marshall, A. 2010. Two-Eyed Seeing: Traditional Mi’kmaw Knowledge and Co-learning. Public Lecture organized by the Atlantic Policy Congress of First Nations Chiefs Secretariat. Mount Saint Vincent University, Halifax, NS, 28 October 2010. [Invited Oral Presentation]

Marshall, A. 2010. Traditional Mi’kmaw Knowledge for Fisheries Resource Management. Aboriginal Fisheries Workshop organized by: The North Shore Tribal Council. Moncton, NB, 22 September 2010. [Invited Oral Presentation]

Marshall, A. 2010. Two-Eyed Seeing. Inaugural Conference - The Ecological Community - of the Association for Literature, Environment, and Culture in Canada (ALECC). Cape Breton University, Sydney, NS. 19-22 August 2010. [Invited Plenary Panel Speaker]

Marshall, A. 2010. Two-Eyed Seeing: water – our life blood. First Nations Water Workshop organized by: Centre for Indigenous Environmental Resources (CIER); Simon Fraser University’s Adaptation to Climate Change Team (ACT); and Bob Sanford, Chair of the Canadian Partnership Initiative of United Nations International “Water for Life” Decade. Toronto, ON, 15-16 July 2010. [Invited Oral Presentation; attendance precluded by family circumstances]

Marshall, A. 2010. Connecting with Evidence and Other Ways of Knowing - Two-Eyed Seeing and Traditional Mi’kmaw Knowledge. Making Connections for Public Health Practice, Policy and Research. National Collaborating Centres for Public Health Summer Institute 2010, session held in conjunction with PHIRNET (Population Health Intervention Research Network). Winnipeg, MB, 28-30 June 2010. [Invited Panel Presentation]

Marshall, A., and Bartlett, C. 2010. Environmental Sustainability and Two-Eyed Seeing. College of Sustainability, Dalhousie University, Halifax, NS, 23 September 2010. [Invited Oral Presentation]

Marshall, A., and Bartlett, C. 2010. Integrative Science and Two-Eyed Seeing; in the spirit of cultural humility. Workshop to “Integrate Cultural Competency and Cultural Safety into Curriculum” organized by Nova Scotia Community College School of Health and Human Services, and St. Francis Xavier University School of Nursing. Millbrook, NS, 12 March 2010. [Invited Oral Presentation]

Marshall, A., Bartlett, C., and Marshall, M. 2010. Ta'ntelo'lti'k – Mi’kmaq Knowledge and Two-Eyed Seeing. Time and A Place Conference: Environmental Histories, Environmental Futures, and Prince Edward Island. University of Prince Edward Island, Charlottetown, PEI, 13-18 June 2010. [Invited Keynote Presentation]

Marshall, A., Marshall, M., and Iwama, M. 2010. Approaching Mi’kmaq Teachings on the Connectiveness of Humans and Nature. In: S. Bondrup-Nielsen, K. Beazley, G. Bissix, D. Colville, S. Flemming, T. Herman, M. McPherson, S. Mockford, and S. O’Grady (Eds). 2010. Ecosystem Based Management: Beyond Boundaries. Proceedings of the Sixth International Conference of Science and the Management of Protected Areas, 21–26 May 2007, Acadia University, Wolfville, Nova Scotia. Science and Management of Protected Areas Association, Wolfville, NS. [Article]

**2009**

Hatcher, AM., Bartlett, C.M., Marshall, M., and Marshall, A. 2009. Two-Eyed Seeing in the classroom environment: concepts, approach and challenges. Canadian Journal of Science, Mathematics, and Technology Education, 9(3): 141-153. [Article, peer-reviewed]

Hatcher, A., Bartlett, C., Marshall, M., and Marshall, A. 2009. Two-Eyed Seeing: across-cultural science journey. Green Teacher, 86: 3-6. [Article]

Iwama, M., Marshall, M., Marshall, A. and Bartlett, C. Two-Eyed Seeing and the Language of Healing in Community-Based Research. Canadian Journal of Native Education, 32: 3-23. [Article]

Marshall, A. 2009. Two-Eyed Seeing. Second International Dialogue on Underwater Munitions, Honolulu, Hawaii. 5-27 February 2009. [Invited Keynote]

Marshall, A. and Marshall, M. 2009. Two-Eyed Seeing - Native Wisdom as our Climate Changes. Tatamagouche Centre. Tatamgouche, NS, 2 May 2009. [Invited Day-Long Gathering Presentation and Discussion]

Marshall, A. and Kavanagh, S. 2009. Two-Eyed Seeing. Memorial High School Mi’kmaq Studies Courses and Students. Sydney Mines, NS, 21 April 2009. [Invited Presentation]

Marshall, A. and Bartlett, C. 2009. Integrative Science and Two-Eyed Seeing. Consultation Workshop re Land-based Education for Inuit Youth and College Students, organized by Environmental Education Specialist (Sharina Dodsworth) of the Department of Environment, Government of Nunavut. Iqaluit, NU, 7-8 April 2009. [Invited and Sponsored Participants and Presenters]

Marshall, A. and Bartlett, C. 2009. Integrative Science and Two-Eyed Seeing. Life Long Learning – from Youth to Elder; conference on Aboriginal Education, organized by Atlantic Aboriginal Economic Development Integrated Research Program (AAEDIRP) of the Atlantic Policy Congress of First Nation Chiefs Secretariat. Fredericton, NB, 23-25 March 2009.

[Lead Plenary Presentation]

Marshall, A. and Bartlett, C. 2009. Co-Learning re “Talking and Walking Together” of Indigenous and Mainstream Sciences. Regular monthly meeting of the Collaborative Environmental Planning Initiative (CEPI) for the Bras d’Or Lakes ecosystem. Membertou First Nation, NS, 19 February 2009. [Requested Presentation]

Marshall, A. and Bartlett, C. 2009. Integrative Science, Two-Eyed Seeing, and forestry – Lessons Learned, Part 2. Indigenous Science Funding Stream Workshop 2. Co-hosted by BC Government Forest Investment Account – Forest Science Program and First Nations Forestry Council. Richmond, BC, 6-9 January 2009. [Oral Presentation, as Invited Speaker, and Invited Workshop Participant]

Marshall, A., Harris, P., Bartlett, C., and Marshall, M. 2009. Spirits of Health: co-learning stories of interconnectiveness. Cultural Knowledge and the Healthy Society: a research and innovation summit, organized by the Ontario College of Art and Design (OCAD). OCAD, Toronto, ON, 23-25 November 2009. [Invited Keynote Presentation]

Marshall, A., Hatcher, A., and Bartlett, C. 2009. Integrative Science and Two-Eyed Seeing. Teachers’ Workshop of the Cape Breton-Victoria Regional School Board. Sydney, NS, 30 November 2009. [Oral Presentation, as Invited Speaker]

Marshall, A., Marshall, M., and Hatcher, A.M. 2009. Aboriginal Economic Development in the Atlantic: Balancing Culture and Environmental Sustainability. Research Workshop organized by Patricia Doyle-Bedwell (Dalhousie University) and the Atlantic Aboriginal Economic Development Integrated Research Program (AAEDIRP) of the Atlantic Policy Congress of First Nation Chiefs Secretariat. Halifax, NS, 6 March 2009. [Invited Workshop Participants]

**2008**

Bartlett, C.M. and Marshall, A. 2008. The Mi’kmaq “healing tense” and Two-Eyed Seeing. Health Canada Senior Management National Meeting, Membertou First Nation, NS; 17 June 2008. [Oral Presentation, as Invited Speaker]

Bartlett, C., and Marshall, A. 2008. Integrative Science and Two-Eyed Seeing. Royal Roads University, Vancouver, BC, 2 December 2008. [Invited Oral Presentation]

Bartlett, C., and Marshall, A. 2008. Integrative Science and Two-Eyed Seeing for International Year of Astronomy – Canadian Aboriginal Component: Mi’kmaw Night Sky Story of Muin and the Seven Bird Hunters. Aboriginal Astronomy Workshop, Office of Indigenous Affairs, University of Victoria. Dunsmuir Lodge, Victoria, BC, 1 December 2008. [Invited Oral Presentation]

Bartlett, C.M., Marshall, A. and Marshall, M. 2008. Facilitating the “talking and walking together” of indigenous and mainstream sciences. Annual International Conference of the Wildlife Disease Association, Edmonton, AB, 3-8 August 2008. [Refereed Oral Presentation (by Bartlett)]

Marshall, A. 2008. Two-Eyed Seeing and Eels. Aboriginal SARA Interdepartmental Working Group – Aboriginal (Traditional) Knowledge Workshop: Using the Example of the American eel. Moncton, NB, 18-19 November 2008. [Oral Presentation, as Invited Speaker and Invited Workshop Participant]

Marshall, A. 2008. Mi’kmaq Elders’ Council and the CEPI process. Elders’ Workshop for Collaborative Environmental Planning Initiative. Wagmatcook First Nation, NS, 6-7 November 2008. [Oral Presentation and Workshop Participant]

Marshall, A. 2008. Netukulimk and natural resources conservation. First Nations’ Social Studies Teachers In-service. Eskasoni First Nation, NS, 24 October 2008. [Oral Presentation]

Marshall, A. 2008. SARA (Species at Risk Act): Aboriginal Traditional Knowledge, regional workshop. Halifax, NS, 21-23 October 2008. [Oral Presentation, as Invited Speaker]

Marshall, A. 2008. Elders Need to Share to be Healthy. Aboriginal Experiences in Aging Symposium, Setting Research and Policy Priorities, Saskatoon, SK, 17-19 September 2008. [Oral Presentation, as Invited Speaker]

Marshall, A. 2008. Two-Eyed Seeing for “What is Evidence and How Do You Apply It?”. National Collaborating Centres’ Summer Institute; Kelowna, BC, 5-9 August 2008. [Invited Panel Speaker (precluded by family circumstances)]

Marshall, A. 2008. First Nations’ perspectives on forestry. Sustainable Hardwood Management in Today’s Acadian Forests; conference organized by UINR, Membertou First Nation, Nova Scotia, 8-9 July 8-9. [Invited Speaker]

Marshall, A. 2008. Health and the Environment: the perspective of an Aboriginal Elder. Public Health in Canada: reducing health inequalities through evidence and actions; Canadian Public Health Association Annual Conference, Halifax, Nova Scotia, 1-4 June 2008. [Invited Oral Presentation]

Marshall, A. 2008. Studio on sustainable Mi’kmaq village design. Professional Graduate Students’ Program, Faculty of Architecture and Planning, Dalhousie University, Halifax, NS, May – June 2008. [Consultant to Program]

Marshall, A. 2008. Elders’ Groups and Cultural Continuity. Dakota Sacred Sites Symposium, University of Minnesota, 4-5 April 2008. [Keynote Speaker]

Marshall, A. and Bartlett, C. 2008. Integrative Science, Two-Eyed Seeing, and forestry – Lessons Learned, Part 1. Indigenous Science Funding Stream Workshop 1, for BC Forest Investment Account – Forest Science Program, BC Government and First Nations Forestry Council. Richmond, BC, 3-5 December 2008. [Oral Presentation, as Invited Speaker, and Invited Workshop Participant]

Marshall, A. and Bartlett, C. 2008. Integrative Science and Two-Eyed Seeing. Royal Roads University, noon hour guest presentation. Vancouver, BC, 2 December 2008. [Oral Presentation, as Invited Speaker]

Marshall, A. and Bartlett, C. 2008. Integrative Science and Two-Eyed Seeing for International Year of Astronomy – Canadian Aboriginal Component: Mi’kmaw Night Sky Story of Muin and the Seven Bird Hunters. Aboriginal Astronomy Workshop, Office of Indigenous Affairs, University of Victoria. Dunsmuir Lodge, Victoria, BC, 1 December 2008. [Oral Presentation, as Invited Speaker, and Invited Workshop Participant]

Marshall, A. and Kavanagh, S. 2008. Two-Eyed Seeing and Eels. Eastern Ontario and Western Quebec Aboriginal Workshop. SARA and ESA Issues including: Aboriginal Traditional Knowledge Sharing. American Eel SARA Listing, and Draft National Management Plan. Ottawa, ON, 22-24 November 2008. [Oral Presentation, as Invited Speaker and Invited Workshop Participant]

Marshall, A. and Kavanagh, S. 2008. SARA (Species at Risk Assessment): Aboriginal Traditional Knowledge, national workshop. Toronto, ON, 28-30 October 2008.

[Oral Presentations, as Invited Speakers, and Invited Workshop Participant]

- Session: ATK and SARA Recovery Planning – How can/should ATK be incorporated into SARA recovery documents? Opened by Marshall and Kavanagh sharing experiences.
- Session: Integrative Science – A Two-Eyed Seeing Approach. Session by Marshall and Kavanagh.

Marshall, M. 2008. Health and healing - death and dying: women’s roles within. Workshop training materials for cultural sensitivity and cultural humility; for health organization. [Article]

**2007**

**Bartlett, C., Kavanagh S., Lefort, N., Marshall, A. and Marshall M**. 2007. Strengths in Our Differences. Fifth Annual Cape Breton Health Research Symposium: Human Health and the Environment, Cape Breton University, April 26, 2007. [Poster]

**Bartlett, C.M., Marshall, A. and Marshall, M. 2007.** Integrative Science:  Enabling Concepts within a Journey Guided by Trees Holding Hands and Two-Eyed Seeing. Two-Eyed Seeing Knowledge Sharing Series, Manuscript No. 1. Institute for Integrative Science & Health, Cape Breton University, Sydney, Nova Scotia, Canada, May 2007. [on-line article available at: <http://www.integrativescience.ca/> under “articles – 2007”]

Iwama, M., Marshall, M., Marshall, A., Mendez, I, and Bartlett, C. 2007. I Got It From An Elder; conversations in healing language. Gaspereau Press, Kentville, Nova Scotia. [Publication]

**Kavanagh, S., Bartlett, C., Lefort N., Marshall, A. and Marshall M.** Picturing Human Health and the Environment. Fifth Annual Cape Breton Health Research Symposium: Human Health and the Environment, Cape Breton University, April 26, 2007. [Poster]

Marshall, A. 2007. Netukulimk. Two Countries – One Forest Conference, Montreal, QC. November 2007. [Invited Speaker]

Marshall, A. 2007. Two-Eyed Seeing and some companion thoughts. ManOMin Watershed Conference; “Seeing with both eyes: Balancing Traditional Aboriginal Knowledge and Contemporary Science”, a conference hosted by International Falls, Minnesota, April10-12, 2007. [Presentation, as Invited Keynote Speaker (precluded by family circumstances)]

Marshall, A. 2007. Two-Eyed Seeing from the perspective of a Mi’kmaq Elder. Two-Eyed Seeing Science Education for Children and Youth Workshop, Cape Breton University, May 24, 2007. [Oral Presentation, as Key Speaker]

Marshall, A. 2007, Two-Eyed Seeing: a useful and powerful metaphor for learning about, and collaborating within, the common ground for Aboriginal and Western Sciences. Fifth Annual Cape Breton Health Research Symposium: Human Health and the Environment, Cape Breton University, April 26, 2007. [Invited Keynote Speaker]

Marshall, A. 2007. Two-Eyed Seeing and Mi’kmaq Knowledge. Evidence and the Social Determinants of Health: Assessing the Possibilities, a Think Tank hosted by the National Collaborating Centre on the Determinants of Health, Halifax, Nova Scotia, March 7, 2007. [Participant, as Invited Knowledge Elder]

Marshall, A. 2007. Two-Eyed Seeing and Mi’kmaq Knowledge. Indigenous Knowledge, a Dialogue Circle hosted by the National Collaborating Centre for Aboriginal Health, at the First Nations’ Long House at the University of British Columbia, Vancouver, British Columbia, February 8, 2007. [Participant, as Invited Knowledge Elder]

Marshall, A. 2007. Elder Knowledge Advisor for manuals writing team for RCMP Aboriginal Shield national school outreach program, within March 2006 contractual bid via Epona Communications Ltd (an Aboriginal business); writing involved 2 manuals each for Grades 4, 6, and 8 (student plus facilitator), plus manual for trainer and manual for cultural resources. [Publication (RCMP manuals)]

**2006**

**Bartlett, C.M., Iwama, M, and Marshall, A**. 2006.  Two-eyed seeing … with strengths of Iindigenous and Western scientific knowledges.  Acadia University Science Café, Wolfville, Nova Scotia, 6 November 2006. [Oral Presentation]

Marshall, A. 2006. Studio on Sacred Site Preservation. Department of Architecture, University of Minnesota, December. [Guest Reviewer]

Marshall, A. 2006. Cultural Issues to Pisquepaq Design Group for Pictou Landing First Nation Health Centre. [Consultancy]

**2005**

**Bartlett, C.M., and Marshall, A.** 2005. “Spirit of the East” and “Two-Eyed Seeing” (conference keynote address).  Canadian Aboriginal Science and Technology Society Conference 2005 (7^th^ National), Membertou-Sydney, Nova Scotia, September 22-44, 2005. [Oral Presentations]

**Marshall, A.** 2005. The science of humility.  In:  New horizons of knowledge; Proceedings for: Te Toi Roa - Indigenous Excellence, World Indigenous Peoples’ Conference on Education, Hamilton Aotearoa New Zealand, November 27 - December 1, 2005. [Oral Presentation plus Publication in Conference Proceedings]

Marshall, A. and Kelloway, M. Integrative Science. Canadian Council on Learning National Workshop for Aboriginal Learning Knowledge Centre, Ottawa, Ontario, November 2005. [Dialogue Participant]

Marshall, M. 2005. On Tribal Consciousness – The Trees That Hold Hands. Te Tol Roa - Indigenous Excellence, World Indigenous Peoples’ Conference on Education. Hamilton Aotearoa, New Zealand, 27 November - 1 December 2005. [Oral Presentation]

Marshall, M. 2005. On Tribal Consciousness – The Trees That Hold Hands. In: New horizons of knowledge section; Proceedings for: Te Tol Roa - Indigenous Excellence, World Indigenous Peoples’ Conference on Education. Hamilton Aotearoa, New Zealand, 27 November -1 December 2005. [Article]

**2004**

Marshall, A. 2004. Traditional Ecological Knowledge. Watershed Workshop, Saskatchewan, November, 2004. [Oral Presentation, as Invited Participant]

Marshall, A. 2004. Symposium on First Nations’ Culture and Environmental Values. University of Minnesota, October. [Keynote Speaker]

**2002**

Marshall, A. 2002. Development of Native Culture-centered Post-secondary Education. Cultural Exchange with Cree Nation of Alberta. Blue Quills College, Blue Quills, AB, September. [Visiting Speaker]

**1990’s - 2000’s**

Bartlett, C., Marshall, M., Marshall, A. and others. Development of proposal document for “Integrative Science” as a new, 4 year undergraduate science degree program, plus proposal documents and curricula for new, accompanying MS~~I~~T science courses. Cape Breton University, Sydney, NS. Details available at:

<http://www.integrativescience.ca/Program/>

<http://www.integrativescience.ca/Origins/>

<http://www.integrativescience.ca/Origins/1990/>

<http://www.integrativescience.ca/Origins/2000/>

<http://www.integrativescience.ca/Origins/2010/>
