# Supplementary material for: How is Etuaptmumk/Two-Eyed Seeing characterized in Indigenous health research? A scoping review
Source: PLoS One. 2021 Jul 20;16(7):e0254612. doi: 10.1371/journal.pone.0254612 (PMC8291645; doi:10.1371/journal.pone.0254612)
Supplement: S1 Table — Sources of information and number of results retrieved. (DOCX) [file pone.0254612.s003.docx]

**S1 Table. Search Strategies.** Electronic databases and websites searched and results retrieved.

| **Bibliographic databases**  1^st^ search conducted March 8, 2019  Query: “Two Eyed Seeing” OR Etuaptmumk  Limit: Published in 2004 and onward, in English language  2^nd^ search conducted on May 12, 2020  Query: “Two Eyed Seeing” OR Etuaptmumk  Limit: Published between March 2019 and May 2020, in English language  EMBASE and ProQuest: 2019-2020, English | | |
| --- | --- | --- |
| Databases | 1st search results | 2nd search results |
| PubMed MEDLINE | 18 | 14 |
| Academic Search Premier (EBSCOhost) | 17 | 21 |
| PsycINFO (EBSCOhost) | 12 | 6 |
| CINAHL (EBSCOhost) | 10 | 11 |
| Bibliography of Native North American (EBSCOhost) | 6 | 1 |
| EMBASE | 18 | 15 |
| ProQuest Theses and Dissertations | 119 | 11 |
| **Other sources**  Searched in March 2019 and May 2020 by using keywords “Two Eyed Seeing” and Etuaptmumk | | |
| Name | 1^st^ search | 2^nd^ search |
| Institute for Integrative Science & Health (IISH) website | 4 | 0 |
| Reference lists | 1 | 11 |
| International Indigenous Policy Journal | 0 | 4 |
