# Supplementary material for: How is Etuaptmumk/Two-Eyed Seeing characterized in Indigenous health research? A scoping review
Source: PLoS One. 2021 Jul 20;16(7):e0254612. doi: 10.1371/journal.pone.0254612 (PMC8291645; doi:10.1371/journal.pone.0254612)
Supplement: S2 Table — Year of publication, study participants, geographical location, research design and aim of study. (DOCX) [file pone.0254612.s004.docx]

# S2 Table. Characteristics of articles describing Two-Eyed Seeing by original authors.

| **Author year** | **Study participants and geographical location** | **Research design** | | **Aim of study** |
| --- | --- | --- | --- | --- |
|  |  | **Empirical: TES; CBPR, CBR, CBPA, PR, or PAR; QT or QL** | **Non-empirical** |  |
| Bartlett2007^4^α | Participants: N/A  Location of relevancy: Nova Scotia, CAN | N/A | Discussion of IS program | Describe bringing together Indigenous and Western sciences and ways of knowing to develop a post-secondary science program. |
| Bartlett 2015^34^† | Participants: N/A  Location of relevancy: Nova Scotia, CAN | N/A | Discussion of IS program | Partial telling of a story of the meeting of Indigenous and Western perspectives and the emergent understandings. |
| *Bartlett 2012^5^‡ | Participants: N/A  Location of relevancy: Nova Scotia, CAN | N/A | Discussion of IS program | Historical overview of weaving Indigenous knowledge and mainstream science in a collaborative, co-learning journey called IS. |
| Bartlett 2014^35^€ | Participants: N/A  Author location: CAN | N/A | Discussion of TES in education | Discuss the importance of Indigenous and non-Indigenous collaboration in education, under TES guiding principle. |
| Hatcher 2009a^36^‡ | Participants: N/A  Location of relevancy: Nova Scotia, CAN | N/A | Discussion of TES in education | Outline concepts and approaches for teaching IS using TES guiding principle and discuss challenges to overcome. |
| Hatcher 2009b^104^€ | Participants: N/A  Location of relevancy: Nova Scotia, CAN | N/A | Discussion of IS program | Present concepts and lessons that lie in the common ground between western and Indigenous sciences and ways of knowing. |
| Iwama 2009^2^‡ | Participants: N/A  Location of relevancy: Unama’ki, Mi’kmaki, Nova Scotia, CAN | N/A | Discussion of TES in Institute for IS | Introduce a community participation model of research and teaching intended to restore health and university a welcoming place. |
| *Marshall 2018^28^€ | Participants: N/A  Author location: CAN | N/A | Discussion of TES in education | Emphasizes the importance of exchange stories because this is at the root of developing relationships. |
| Marshall 2015^3^† | Participants: N/A  Author location: Mi’kma’ki, Nova Scotia, CAN | N/A | Discussion of TES in medical/  health education | Discuss how TES highlights the benefits of medical science and Indigenous knowledges for teaching medical and health science students. |

*The term Etuaptmumk has been used in the article. Grey literature and journal articles were included: book chapter†, journal article‡, magazine article€ and unpublished manuscriptα.

Abbreviations: Canada = CAN; Community-based participatory research = CBPR; Community-based participatory action methodology = CBPA; Community-based research = CBR; Integrative Science = IS; Not applicable = N/A; Participatory action research = PAR; Participatory research = PR; Qualitative = QL; Quantitative = QT; Social determinants of health = SDOH; Two-Eyed Seeing = TES
