# Supplementary material for: How is Etuaptmumk/Two-Eyed Seeing characterized in Indigenous health research? A scoping review
Source: PLoS One. 2021 Jul 20;16(7):e0254612. doi: 10.1371/journal.pone.0254612 (PMC8291645; doi:10.1371/journal.pone.0254612)
Supplement: S3 Table — Year of publication, study participants, geographical location, research design and aim of study. (DOCX) [file pone.0254612.s005.docx]

# **S3 Table. Characteristics of articles describing Two-Eyed Seeing by new authors.**

| **Author year** | **Study participants and geographical location** | **Research design** | | **Aim of study** |
| --- | --- | --- | --- | --- |
|  |  | **Empirical: TES; CBPR, CBR, CBPA, PR, or PAR; QT or QL** | **Non-Empirical** |  |
| Antonio 2019^15^§ | Participants: Navajo wool rug weavers, aged ≥18 years  Research locations: In or near the Navajo Nation (community chapter affiliations: Shiprock, St. Michaels, Ganado, Chinle, Teec Nos Pos), US | QL: Indigenous methodologies and lens, semi-structured interviews, narrative inquiry, arts-informed visual analysis | N/A | Determine how sustainability might be conveyed through the worldviews, experiences, and practices of some Navajo wool rug weavers. |
| Auger 2016^37^‡ | Participants: N/A  Location of relevancy: CAN; US | Meta-synthesis of QL research | N/A | Describe and interpret qualitative research on cultural continuity for North American Indigenous Peoples. |
| Baydala 2016^33^‡ | Participants: Four Maskwacis (Cree) Nations elementary and junior high school students, Elders, parents, school personnel, program facilitators  Research location: Alberta, CAN | CBPR  QT: Sequential longitudinal cohort design, questionnaires; QL: Focus groups | N/A | Culturally adapt the Life Skills Training program to reflect the culture, language, and visual images of the Maskwacis community, deliver it in Maskwacis schools, and evaluate its impact. |
| Benoit 2019^34^‡ | Participants: Cis- or trans-women self-identifying as First Nation, Métis or Inuk, aged ≥18 years  Research locations: Toronto & Thunder Bay, Ontario, CAN | CBR, TES  QT: Questionnaire; QL: Sharing circles or interview | N/A | Present the discourse on racism experiences of Indigenous women living in two urban Canadian cities. |
| Black 2016^35^‡ | Participants: N/A  Location of relevancy: CAN | N/A | Policy analysis | Increase Indigenous participation in the decision-making process on environmental issues and propose a framework to meet this outcome. |
| Blangy^36^ 2018‡ | Participants: N/A  Location of relevancy: Nunavik, Québec, CAN | N/A | Description of OHMi-Nunavik research program | Introduce the OHMi Nunavik Special Issue, present the OHMi Nunavik methodology and review ongoing projects and their contributions to Nunavik community wellbeing. |
| Blind 2017^39^§ | Participants: Indigenous people with neurological condition or caring for someone with a neurological condition, health care providers, and Knowledge Keepers  Research location: 4 provinces, 1 territory, CAN | TES  QL: Indigenous methodology, interviews, research circles | N/A | Determine the scope of 14 under-studied neurological conditions in Canada (incidence, prevalence, comorbidities), their risk factors and impacts on people living with them, their families, caregivers, and communities; and existing health services effectiveness. |
| *Bruner 2019^37^‡ | Participants: First Nations, Métis or Inuit youth, aged 15-25 years  Research locations: 7 urban & 3 reservation/rural communities from British Columbia, Nunavut to Newfoundland and Labrador, CAN | QL: Sharing circles | N/A | To understand Indigenous youth development within the context of sport and physical activity through the voices, stories, and experiences of Indigenous youth. |
| Butler 2018^38^‡ | Participants: N/A  Location of relevancy: University of Saskatchewan, Saskatchewan, CAN | N/A | Discussion of Indigenous engagement | Describe implementing the "learn where you live" delivery model at the universities College of Nursing by the Strategist for Outreach and Indigenous Engagement. |
| Cabrera 2015^40^‡ | Participants: Talthan community  Research location: British Columbia, CAN | CBPR  QL: Focus group, interviews | N/A | Explore the impact and interplay of medicalization with the Nation’s knowledge and approaches to wellness in relation to early onset familial Alzheimer disease. |
| Carter 2017^41^‡ | Participants: First Nations men in Toronto  Research location: Ontario, CAN | TES  QL: Narrative methodology, interviews | N/A | Counter negative Aboriginal men stereotypes by exploring positive Aboriginal identity narratives and explore ways health care providers deliver culturally safe care and support Aboriginal clients’ wellbeing. |
| Castleden 2017^43^‡ | Participants: Community-based and academic researchers conducting water research using Western and Indigenous knowledge systems  Research locations: CAN | QL: Semi-structured interview | N/A | Examine how different ontologies of water are part of different ways of relating to water and how water has an important effect on the allocation and enactment of roles in collaborative research partnerships. |
| *Chatwood 2015^42^‡ | Participants: N/A  Location of relevancy: Fly-in lodge in northern CAN | N/A | Discussion of research method | Describe a collaborative mixed method study with Indigenous knowledge used by researchers and Indigenous knowledge holders on Indigenous values underlying health systems stewardship. |
| Clark 2012^45^§ | Participants: Urban Inuit, electronic health program stakeholders  Research location: Manitoba, CAN | TES  QL: Focus groups, interviews | N/A | Empower Inuit to attain self-determination by managing their personal health data; and develop empowering & culturally safe practices used Manitoba Urban Inuit Association. |
| Clark 2014^46^‡ | Participants: Inuit recommended or endorsed by the Manitoba Urban Inuit Association, electronic health program managers, Inuk Elder  Research location: Manitoba, CAN | TES  QL: Focus groups, interviews | N/A | Offer best practice recommendations for how to communicate personal health information services to Manitoba Inuit and increase their awareness of issues related to electronic health information within the healthcare system. |
| Crooks 2018^47^‡ | Participants: MHFAFN facilitators and participants from First Nations communities, organizations in urban centres, & rural communities  Research location: 4 provinces, CAN | CBPR, TES  QT: Feasibility study with retrospective pre-post evaluation study design, survey; QL: Interviews | N/A | Undertake a feasibility study of the Mental Health First Aid First Nations (MHFAFN) course to assess its acceptability, cultural adaptation, and preliminary participant outcomes. |
| *Denny 2016^48^‡ | Participants: N/A  Location of relevancy: Nova Scotia, CAN | N/A | Discussion of salmon governance | Compare conservation concepts in Atlantic salmon management from a non-Aboriginal state perspective with a Mi’kmaq perspective to develop Atlantic salmon governance initiatives. |
| *Fayed 2018^49^‡ | Participants: N/A  Author location: British Columbia, CAN | N/A | TES  Reconciliation-based analysis, literature review | Expose the colonial etiology of hepatitis C infection in Canada and propose potential anti-colonial approaches to hepatitis C wellness and health care for Indigenous people. |
| Fontaine 2019^50^‡ | Participants: First Nations women, aged ≥65 years with a medically diagnosed condition or been a caregiver for someone with a heart condition  Research locations: Winnipeg and Northern communities, Manitoba, CAN | QL: Digital storytelling and learning circles | N/A | Identify concepts, language, and experiences of heart health among First Nations women. |
| Fornssler 2018^51^† | Participants: N/A  Author location: Saskatchewan, CAN | N/A | Reflections | Thoughts, concerns, responses, and visioning of Indigenous and non-Indigenous research team members with challenges on working collaboratively on a decolonizing project. |
| *Gray 2019^52^‡ | Participants: First Nations communities  Research location: New Brunswick, CAN | QT: Contaminant screening via analytical chemistry | N/A | Assess spatial and temporal environmental contamination in muskrat root; assess human health risk by consumption; and provide a baseline against which future monitoring could be compared with. |
| Hall 2015a^53^‡ | Participants: N/A  Research locations: 12 First Nations treatment centres across CAN | N/A | Discussion of TES in a research study | Apply TES in the 1^st^ year of a 3-year study on the effectiveness of cultural interventions in First Nations alcohol and drug treatment in Canada. |
| Hall 2015b^54^† | Participants: N/A  Research locations: 12 community treatment centres in CAN | N/A | Interview transcription | Describe trans-disciplinarity, its relationship to Indigenous ways of knowing; and using TES in addictions research with Indigenous peoples. |
| Hatala 2017^55^‡ | Participants: Plains Cree and Métis youth, aged 15-25 years  Research location: Saskatoon, Saskatchewan, CAN | TES  QL: Interviews | N/A | Determine how youth experience or orient themselves toward conceptions of time; the impact of systemic social inequities and historical or contemporary traumas; and individual strategies and interpersonal contexts that support processes of resilience and wellbeing. |
| *Hatala 2020^56^‡ | Participants: Plains Cree and Métis youth, aged 15-25 years  Research location: Inner city neighbourhoods, Saskatoon, Saskatchewan, CAN | TES  QL: Photovoice, interviews | N/A | Explore how connections to land and nature could inform public health interventions for contemporary Indigenous young people experiencing various social inequities within their urban environments. |
| Heath-Engel 2016^57^§ | Participants: Indigenous and alternative health practitioners  Research location: Ontario, CAN | TES  QL: Indigenous methodology, interviews | N/A | Deconstruct the term ‘psychosis’ by exploring its origins, a personal narrative of this phenomenon, and perspectives from around the world on experiences in this category. |
| Hinds 2014^58^§ | Participants: Aboriginal students, staff, and Elders at 3 colleges  Research location: Ontario, CAN | TES  QL: Information extraction from websites, interviews, narrative and phenomenology | N/A | Determine what strategies and programs help Aboriginal students succeed at postsecondary institutions. |
| *Hovey 2017^59^‡ | Participants: N/A  Location of relevancy: Kahnawake Mohawk Territory, Québec, CAN | N/A | Discussion of TES in a research study | Bring together a health promotion research team to understand shared Indigenous & non-Indigenous perspectives of diabetes prevention and health promotion in an Indigenous community; show how allied health promotion and Indigenous researchers work together through TES; and describe becoming TES researchers. |
| Hunt 2018^60^‡ | Participants: First Nations youth playing recreational hockey, their parents, and coaches  Research location: First Nations reserve, Northern Ontario, CAN | TES  QT: Survey | N/A | Begin to understand concussion in youth hockey in a First Nations community in Canada and to determine the impact of a novel concussion education workshop. |
| Hutt-MacLeod 2019^16^‡ | Participants: N/A  Location of relevancy: Eskasoni, Nova Scotia, CAN | N/A | Review of a mental health service | Describe implementing the ACCESS OM objectives for youth mental health service transformation within a pre-existing Fish Net Model of transformative youth mental healthcare service in Eskasoni. |
| Knapp 2013^61^€ | Participants: N/A  Author location: N/R | N/A | Discussion of TES in education | Suggest how teachers could help their students nurture a sense of place and awareness of their communities. |
| Kurtz 2017^62^‡ | Participants: Indigenous nursing new graduates, employers, educators, and policy makers  Research location: Western CAN | TES  QL: Indigenous methodology: interviews, one day forum, talking circles | N/A | Examine Indigenous nurses’ transition from education to practice and their career development experiences; increase understanding retention issues for Indigenous nurses in the workplace. |
| Latimer 2014^63^‡ | Participants: N/A  Author location: CAN | N/A | Literature review | Raise awareness and create new understandings in pediatric pain knowledge to reflect the best of Indigenous and Western perspectives on how to enhance health care encounters, reduce the hurt & increase Aboriginal children wellbeing. |
| Latimer 2018^64^‡ | Participants: First Nation youth aged 8-17 years  Research locations: 4 First Nations communities (3 Mi'kmaw and 1 Wolastoqey regions), NB, NS, PEI; CAN | CBPA, TES  QL: Ethnographic techniques, talking circles, art workshop | N/A | Determine how First Nations youth express pain to inform culturally appropriate assessment and treatment. |
| Lemke 2017^65^‡ | Participants: N/A  Location of relevancy: International | N/A | Literature and methodology review | Explore methodological considerations for research and the underlying structural issues relating to Indigenous Peoples' food systems and nutrition. |
| Manmohan  2018^66^§ | Participants: Hitaču leaders Chiefs, Elders, legislature members, education professionals, emerging leaders  Research location: Yuułuʔiłʔatḥ community of Hitaču, British Columbia, CAN | Collaborative CBR  QL: Interviews, participant observation, focus group, sharing circles | N/A | Define a third space of leadership that weaves Indigenous and mainstream leadership practices; identify skills, knowledge, and abilities that current and future Indigenous leaders need; and highlight high-impact practices to develop third space leadership attributes in emerging leaders. |
| Mantyka-Pringle 2017^67^‡ | Participants: First Nations and Métis communities  Research location: Slave River Delta, Northwest Territories, CAN | TES  QL: Field observations, document reviews, interviews | N/A | Present a TES approach for co-producing and blending knowledge about ecosystem health by using an adapted Bayesian Belief Network for the Slave River and Delta region in Canada's Northwest Territories. |
| Marsh 2015a^68^‡ | Participants: N/A  Location of relevancy: Ontario, CAN | N/A | Literature review | Explore the feasibility of blending Aboriginal healing practices with a Western treatment model to address intergenerational trauma with substance use disorders in Aboriginal peoples using TES. |
| Marsh 2015b^17^‡ | Participants: N/A  Author location: Sudbury, Ontario, CAN | N/A | Review of research process | Describe the research process taken to incorporate TES Indigenous decolonizing methodology into the treatment of intergenerational trauma and substance use disorders in Aboriginal peoples, using the Seeking Safety model. |
| Marsh 2016^18^‡ | Participants: Indigenous males and females of Ojibway, Cree, and Métis heritage; aged 24-68 years; had intergenerational trauma and substance use disorder  Research location: Sudbury, Ontario, CAN | TES  QT: Questionnaires; QL: Sharing circles, semi-structured interviews, discussion meetings | N/A | Explore whether the blending of Indigenous traditional healing practices and the Western treatment model Seeking Safety, used to treat post-traumatic stress disorder and substance use disorders, resulted in a reduction of intergenerational trauma symptoms and substance use disorders. |
| Marsh 2018^69^‡ | Participants: Males and females of Ojibway, Cree, and Métis heritage; aged 24-68 years; had intergenerational trauma and substance use disorder  Research location: Sudbury, Ontario, CAN | TES  QL: Indigenous decolonizing methodology, sharing circles, semi-structured interviews | N/A | Explore the application of the sweat lodge ceremony as a component of Indigenous Healing and Seeking Safety. |
| Martin 2009^70^§ | Participants: Inuit and Métis community members  Research location: South-eastern Labrador community of St. Lewis | TES  QL: Individual & joint storytelling sessions | N/A | Explore how three generations of adults who live in one Labrador Inuit-Métis community experience and understand their relationships to food in a context of global change. |
| Martin 2012^71^‡ | Participants: N/A  Author location: CAN | N/A | Discussion of TES as a research framework | Describe features and principles of Indigenous & Western perspectives and how they might be used to answer pressing questions about Indigenous people and communities’ health. |
| Martin 2017^72^‡ | Participants: Elders, key informants (health care providers, family caregivers, community leaders)  Research locations: Little Saskatchewan First Nation, Manitoba, CAN | TES  QL: Participatory framework, critical ethnography interviews | N/A | describe the merits and challenges of using TES (Elders and typically non-Indigenous decision-makers) to guide a collaborative research project with a First Nation community in Manitoba, Canada devastated by a human-made flood. |
| McKivett 2019^74^‡ | Participants: N/A  Author location: AUS | N/A | Scoping review | Explore the role and impact of the clinical communication process on Aboriginal healthcare provision. |
| McKivett 2020^73^‡ | Participants: N/A  Location of relevancy: AUS | N/A | Discussion on approach to medical education research | Explore the TES approach in the context of Indigenous medical education research and draws on an Australian research project as an example of its application. |
| McMillan 2016^75^‡ | Participants: N/A  Location of relevancy: Atlantic CAN | N/A | Discussion of fishery management | Review recent developments on fishery policy and management processes where the Mi'kmaw are working to revitalize the place of netukukimk. |
| Peltier 2018^78^‡ | Participants: N/A  Location of relevancy: First Nations communities, Manitoulin Island, Ontario, CAN | N/A | Discussion of TES in a research study | Add to existing qualitative methods by applying TES in the bridging of Indigenous and participatory methodologies. |
| Pictou Landing 2016^44^δ | Participants: Pictou Landing First Nation community  Research location: Pictou Landing First Nation, Nova Scotia, CAN | CBPR, TES  QT: Survey, environmental & contaminant monitoring via analytical chemistry, community mapping | N/A | Conduct environmental and social research to better understand whether effluent from a local pulp and paper mill is making residents of Pictou Land First Nation sick. |
| Rand 2016^79^‡ | Participants: Inuit women aged 18-61 years  Research location: Kugluktuk, Nunavut, CAN | CBPR, TES  QL: Storytelling sessions | N/A | Create a dialogue with Inuit women to address the lack of information to inform programming to improve their sexual health, their families, and their communities in the Canadian Arctic. |
| Rowan 2015^76^‡ | Participants: N/A  Author location: CAN | N/A | Scoping review | Identify and map literature on cultural interventions in addictions treatment; and describe the nature, extent, and gaps in the literature. |
| *Rowett 2018^77^‡ | Participants: N/A  Location of relevancy: Mi’kmaw and Wolastoqey nations | N/A | Discussion of TES as a research approach | Describe Etuaptmumk & its contribution to relationality and relational accountability in future research; review its usage in the literature; and describe the author’s internalized ways of knowing, specifically Mi'kmaw & Wolastoqey knowledges. |
| *Sasakamoose 2017^80^‡ | Participants: First Nations community members; cultural “experts” (Elders, ceremonialists, medicine people or herbalists)  Research location: Saskatchewan, CAN | QL: Indigenous-centred research process, in-depth Indigenous qualitative consultations | N/A | Describe the theoretical development of the Indigenous Cultural Responsiveness Theory (ICRT), a decolonizing model that aims to improve First Nations well-being in Saskatchewan. |
| Shrivastava 2020^81^‡ | Participants: Care providers, administrators, and Cree patients  Research location: Four Eastern James Bay Cree communities in Northern Québec, CAN | PR, TES  QL: Group discussions and individual interviews | N/A | Contribute to the deep understanding of the cultural aspects of the integration of oral health into primary health care at an Indigenous health organization. |
| Sivertsen 2020^82^‡ | Participants: Aboriginal residents in residential aged care and carers  Research location: AUS | QL: Aboriginal centred research, interpretive descriptive approach, semi-structured interviews using a conversational ‘yarning’ style | N/A | Investigate how the Aboriginal residents’ spiritual well-being related to living in residential aged care in South Australia. |
| *Sylliboy 2020^83^‡ | Participants: N/A  Location of relevancy: CAN | N/A | Commentary | Discuss the importance of acknowledging and understanding Indigenous knowledge systems within the health-care system, using TES as an example of how to create space for diverse perspectives. |
| Venner 2018^84^‡ | Participants: American Indians, Alaskan Natives  Location of relevancy: US | QL: One-day meeting | N/A | Describe the results of a meeting of key stakeholders to elicit feedback on the acceptability and uptake of medication assisted treatment for opioid use disorders among American Indians and Alaskan Natives. |
| Victor 2019^85^‡ | Participants: Blackfoot who are homeless and use substances, and key informants (program facilitators and individuals interacting regularly with participants  Research location: Alberta, CAN | TES  QL: General inductive approach to impact and process evaluation, semi-structured interviews | N/A | Evaluate a program for Indigenous people experiencing homelessness and substance misuse. |
| Vukic 2012^19^‡ | Participants: N/A  Location of relevancy: Canada | N/A | Discussion of nursing research method | Explain how CBPR and the principles of OCAP help to integrate TES and ethical space in shaping nursing research to address health priorities with Aboriginal peoples. |
| Vukic 2014^86^§ | Participants: Youth, service providers, teachers, parents, Elders  Research location: Rural Mi’kmaw community, Nova Scotia, CAN | CBPR, TES  QL: Critical Indigenous qualitative inquiry, individual storytelling, talking circles, community forum, participant observation, field notes | N/A | Explore understandings of mental health among rural Mi’kmaw youth and identify potential actions that promote the mental health of youth aged 14 to 19. |
| Vukic 2016^87^‡ | Participants: Mi'kmaw youth and Elders  Research location: rural community (Mimikej as pseudoname), Nova Scotia, CAN | CBPR, TES  QL: Talking circles, community forum, storytelling, field notes, participant observation | N/A | Understand Mi’kmaw youth mental health. |
| Webkamigad 2020^88^‡ | Participants: Health Canada’s First Nations and Inuit Home and Community Care program (FNIHCC) program managers from across Canada and an Elder  Research location: Ontario, CAN | TES  QL: Review of previously collected interview data, review of published literature, environmental scan, on-going meetings with stakeholder | N/A | Outline a unique and innovative process resulting in two evidence-based Indigenous-specific fact sheets on dementia care for caregivers, older adults, people with dementia, and community health care providers. |
| *Whiting 2018^89^‡ | Participants: Eagle Moon Health Office Team including Indigenous and non-Indigenous health consultants/ practitioners (Knowledge Keepers, healers, Elders, administrators)  Research location: Qu’Appelle Health Region, Regina, Saskatchewan, CAN | TES  QL: Exploratory research, interpretive descriptive approach | N/A | Explore the perspectives/experiences of select Saskatchewan health professionals practicing across diverse Indigenous and mainstream healthcare services to understand the concept from their perspectives. |
| *Whitty-Rogers 2013^8^§ | Participants: Mi'kmaq women aged ≥18 years, community members, Elders  Research location: 2 Mi’kmaq communities, Nova Scotia, CAN | PAR, TES  QL: One-on-one conversational interviews, talking circles | N/A | Explore with Mi’kmaq women and community members, including Elders how to begin a conversation on GDM; gather their stories on GDM experiences; explore its implications and meanings; mobilize possible decisions GDM. |
| *Whitty-Rogers 2016^9^‡ | Participants: Mi’kmaq women with GDM  Research location: 2 Mi'kmaq communities, Nova Scotia, CAN | PAR, TES  QL: One-on-one conversational interviews, talking circles | N/A | Explore and gain insight into the experiences of Mi’kmaq women with gestational diabetes mellitus (GDM); explore how SDOH and existing health policies shape these experiences. |
| *Wolfson 2019^90^‡ | Participants: Community leaders, frontline workers, policymakers, and researchers  Research location: Unceded Territories of the Coast Salish Peoples and the Musqueam Territory | Etuaptmumk  QL: One-day meeting and a workshop | N/A | Describe the consensus statement and exemplary FASD prevention programs from Indigenous communities and organizations across Canada to highlight identity, culture, and relationships as central elements of FASD prevention in Indigenous communities. |
| Wright 2019a^91^‡ | Participants: N/A  Research location: Hamilton, Ontario, CAN | N/A | Discussion of TES in research | Describe the application of TES to a community-engaged study aimed at understanding how Indigenous mothers experience using health care to meet the health needs of their infants. |
| Wright 2019b^93^‡ | Participants: Indigenous mothers of infants less than two years of age, providers of early childhood development services  Research location: Ontario, CAN | TES  QL: Interpretive description methodology, semi-structured interviews | N/A | This article is part of a larger study exploring the phenomenon of how Indigenous mothers living in the city of Hamilton, Ontario, experience selecting and using health services to meet the health needs of their infants aged less than two years. |
| Wright 2019c^94^‡ | Participants: Indigenous mothers self‐identifying as First Nations, Métis and/or Inuit, living in Hamilton and caring for an infant younger than two years of age  Research location: Ontario, CAN | TES  QL: Interpretive description methodology, interviews, discussion group | N/A | Develop an understanding of how Indigenous mothers experience selecting and using health services for their infants can assist nurses in improving their access to care. |
| Wright 2019d^95^‡ | Participants: Indigenous mothers and primary care providers  Research location: Ontario, CAN | TES  QL: Interpretive description methodology, semi-structured interviews, discussion group | N/A | Understand how Indigenous mothers—typically responsible for the health of their infants—living in urban areas, experience selecting and using health services to meet the health needs of their infants. |
| Wright 2020^92^‡ | Participants: Indigenous mothers of infants less than two years of age, accessing and using the health care system for their infants  Research location: Ontario, CAN | TES  QL: Interpretive description methodology, interviews, discussion group | N/A | Understand how Indigenous mothers experience accessing and using the health care system for their infants. |

*The term Etuaptmumk has been used in the article. Grey literature and journal articles were included: book chapter†, journal article‡, magazine article€, reportδ, and thesis/dissertation§.

Abbreviations: Australia = AUS; Canada = CAN; Community-based participatory research = CBPR; Community-based participatory action methodology = CBPA; Community-based research = CBR; Integrative Science = IS; Not applicable = N/A; Ownership, control, access, and possession = OCAP; Participatory action research = PAR; Participatory research = PR; Qualitative = QL; Quantitative = QT; Social determinants of health = SDOH; Two-Eyed Seeing = TES; United States = US
