# Supplementary material for: How is Etuaptmumk/Two-Eyed Seeing characterized in Indigenous health research? A scoping review
Source: PLoS One. 2021 Jul 20;16(7):e0254612. doi: 10.1371/journal.pone.0254612 (PMC8291645; doi:10.1371/journal.pone.0254612)
Supplement: S4 Table — Mapping extracted quotations to descriptive categories. (DOCX) [file pone.0254612.s006.docx]

**S4 Table. Thematic analysis of original authors’ descriptions of Two-Eyed Seeing.**

| **Author Year** | **Category 1: Guide for life** | **Category 2: Responsibility for the greater good and future generations** | **Category 3: Co-learning process** | **Category 4: Multiple/diverse perspectives** | | | | **Category 5: Spirit** | **Category 6: Decolonization and self-determination** | **Category 7: Humans being part of ecosystems** |
| --- | --- | --- | --- | --- | --- | --- | --- | --- | --- | --- |
|  |  |  |  | **Respect for multiple realities** | **Perspectives are not static** | **Wholeness/ partiality of knowledge** | **Co-existence of knowledges** |  |  |  |
| Bartlett 2007^4^ | Journey on Mother Earth | “It’s us” consciousness  Motivate people to leave the world a better place |  | Avoid knowledge domination and assimilation  The gift of newness |  | Recognize the distinct and whole nature of knowledges | Bring together different knowledges  Weave back and forth between knowledges (i.e., call upon the strengths within Indigenous or Western sciences in particular sets of circumstances) |  |  |  |
| Bartlett2012^5^ | TES is about life and does not fit into any subject area/ discipline |  |  |  | Always fine-tuning mind and looking for better way |  |  | Spirit is essential for a complete person | Require exploring or undertaking decolonizing work | Biologically derived phrase TES will require radical approaches for human ecology |
| Bartlett 2014^35^ |  |  | Require both parties’ commitment to continual conversation  Imply responsibilities for co-learning | The gift of multiple perspectives |  |  |  |  |  | Relational understanding comes from continual and cyclical commitment to TES |
| Bartlett 2015^34^ | TES is about life and does not fit into any subject area/ discipline | Leave the world a better place |  | Gift of multiple perspectives | Always fine-tuning mind | Acknowledge the distinct and whole nature of knowledges | Not tweaking one knowledge to accommodate the other  Bring together different ways of knowing and see the strengths of Indigenous and Western knowledges | Spirit is essential for a complete person |  |  |
| Hatcher 2009a  ^36^ |  | Motivate people to use all our gifts so we leave the world a better place and do not compromise the opportunities for our youth |  | Avoid knowledge domination and assimilation  Avoid a clash of knowledges |  |  | Recognize the best from both worlds  Intentionally and respectfully bring together different ways of knowing  Concentration on the common ground  Allow the Indigenous Sciences sense of the whole “to dance with” the Western Science sense of the parts | Awaken the spirit and teach you that everything is physical and spiritual |  | Humans are a very small part of the whole |
| Hatcher 2009b^104^ |  |  |  | Avoid a clash or “domination and assimilation” of knowledges |  |  | Take the best from two worlds  Choose Indigenous or Western sciences based on particular sets of circumstances   Weave back and forth between knowledges | Awaken the spirit and teach you that everything is physical and spiritual |  |  |
| Iwama 2009^2^ | Way of living | Work to solve problems in communities  Motivate people to leave the world a better place |  |  |  |  | Not merging knowledge systems  Common ground or overlapping field of shared strengths  Draw together the strengths of knowledges  Bring together different ways of knowing, weave back and forth between knowledges |  |  |  |
| Marshall 2015^3^ | Look for better ways of doing things | For the benefit of all Motivate people to leave the world a better place  Work together for a better and healthier world  Lead to better health outcomes for all |  | Gift of multiple perspectives  Choose to use words that resonate more closely with traditional ways of knowing  Appreciate the wisdom in Traditional Mi’kmaw Knowledge  Help us understand how Traditional Knowledge from an ancient culture can work for a better and healthier world | Traditional Knowledge was never meant to stay static and stay in the past. Rather, we must bring it into the present |  | See the strengths or best in Indigenous and Western knowledges  Bring together different ways of knowing  Determine the benefits both in modern medical science knowledges and in Indigenous knowledges  Draw upon both new technologies and traditional practices |  |  |  |
| Marshall 2018^28^ |  |  | TES brought forward for co-learning  Co-learning is essential to avoid tokenism | The gift of multiple perspective |  |  | Beneficial outcomes are more likely |  |  | TES being relevant to the environment |
