# Supplementary material for: How is Etuaptmumk/Two-Eyed Seeing characterized in Indigenous health research? A scoping review
Source: PLoS One. 2021 Jul 20;16(7):e0254612. doi: 10.1371/journal.pone.0254612 (PMC8291645; doi:10.1371/journal.pone.0254612)
Supplement: S5 Table — Mapping extracted quotations to descriptive categories. (DOCX) [file pone.0254612.s007.docx]

**S5 Table. Thematic analysis of new authors’ descriptions of Two-Eyed Seeing.**

| **Author Year** | **Category 1: Guide for life** | **Category 2: Responsibility for the greater good and future generations** | **Category 3: Co-learning process** | **Category 4: Multiple/diverse perspectives** | | | | **Category 5: Spirit** | **Category 6: Decolonization and self-determination** | **Category 7: Humans being part of ecosystems** |
| --- | --- | --- | --- | --- | --- | --- | --- | --- | --- | --- |
|  |  |  |  | **Respect for multiple realities** | **Perspectives are not static** | **Wholeness/ partiality of knowledge** | **Co-existence of knowledges** |  |  |  |
| Antonio 2019^15^ |  | Intention of advocacy for people and nature |  | Seek to acknowledge and honour multiple perspectives |  | Recognize Indigenous knowledge as a distinct epistemological system existing next to Western (mainstream) science | TES is a collaborative rather than an integration/ amalgamation of knowledge systems |  | Act of decolonization | Embrace and honours an inherent human-nature relationship; suggest that knowledge is relational |
| Auger 2016^37^ | Ontology and way of life |  |  | Honour both Indigenous and Western ontologies and methodologies |  |  | Bring together Western and Indigenous knowledge |  |  | Draw attention to relational aspects of diverse understandings of complicated issues |
| Baydala 2016^33^ |  |  |  | Recognizes diverse perspectives as valid without privileging one viewpoint over another |  |  |  |  |  |  |
| Benoit 2019^34^ | Encourage self-reflection | Be open to new perspectives to reach the most beneficial outcome for those involved | Meaningfully shapes how individuals think about the work they are doing | Encourage individuals to be open to multiple perspectives | Emphasize the transformative nature of knowledge |  |  | Recognize knowledge as alive spiritually and physically |  |  |
| Black 2016^35^ |  |  |  | Give equal consideration to diverse worldviews |  |  | Bring together Indigenous and Western ways of knowing |  |  |  |
| Blangy 2018^36^ |  |  |  | Equal contribution of Indigenous and Western epistemology and knowledge |  |  |  |  |  |  |
| Blind 2017^39^ |  |  |  | See and understand the cultural nuances |  |  | Use both eyes to analyze issues via the strengths of Indigenous and Western ways of knowing  Bring together Indigenous ways of knowing and critical medical anthropology |  | Valuable in looking at the colonial impacts on health and wellbeing |  |
| Bruner 2019^37^ |  |  | Co-learning may provide opportunities to recognize how domination of Western knowledge has limited scientific inquiry | Consider both Indigenous and Western epistemological conceptualizations of the world  Seeing the world via Indigenous worldviews next to European perspectives |  |  | Draw on the strengths of multiple perspectives to create a shared understanding of a topic |  |  |  |
| Butler 2018^38^ |  |  | Examine past actions for a co-learning journey | Respect differing values without placing one as more important than the other |  |  |  |  |  | Reinforce the interconnectedness of both worldviews |
| Cabrera 2015^40^ |  | For the benefit of all |  | Embrace the contributions of different worldviews |  |  | See the strengths of Indigenous and Western knowledges  Weaving back and forth between different knowledges |  |  |  |
| Carter 2017^41^ |  |  |  | Honor multiple viewpoints and respect them as valid |  |  | Interweaving Western and Indigenous ways of knowing |  |  |  |
| Castleden 2017^43^ |  |  |  |  |  | To arrive at a more comprehensive and mutually beneficial understanding | Learning to see the strengths of Indigenous knowledge with one eye, and using the other eye to see with the strengths of Western knowledge |  |  |  |
| Chatwood 2015^42^ |  |  |  | Aim to resolve the inherent conflicts between Indigenous ways and scientific inquiry |  |  |  |  |  |  |
| Clark 2012^45^ |  |  |  |  |  |  |  |  | Lead to outcomes that find spaces for self-determination |  |
| Clark 2014^46^ |  |  |  |  |  |  | Incorporate traditional and Western scientific perspectives  Integrate linguistic components of health from Indigenous and Western knowledges to create a vision with more than one perspective |  | May lead to outcomes that find spaces for Inuit self-determination within existing electronic health information systems |  |
| Crooks 2018^47^ |  |  |  |  |  |  | Bring together the strengths of Indigenous ways of knowing and Western ways of knowing |  |  |  |
| Denny 2016^48^ |  | Co-existence of both perspectives for the benefit of all |  | Interdisciplinary, cross-cultural, and pluralistic approach to addressing wicked problems | Fitting process dependent on the personalities at the table and their receptiveness |  | See the strengths of Indigenous and Western knowledges |  |  |  |
| Fayed 2018^49^ |  |  |  | Respect the great diversity of Indigenous people’s worldviews |  |  | Provide a balancing lens that emphasizes Indigenous health perspectives while upholding and valuing mainstream perspectives |  | Anti-colonial approach to health research and practice  Activate the wider reconciliatory health agenda |  |
| Fontaine 2019^50^ |  |  | Alternative solutions may emerge through respectful listening challenging our ways of knowing and caring for our hearts and health | Adopt a stance of seeing and respectfully considering multiple perspectives  Recognise multiple epistemologies exist rather than positioning them as dichotomous  Understand that First Nation knowledges deserve space in health research and the world generally  Recognise that Indigenous conceptions of heart health and traditional medicines are legitimate for challenging hegemonic devaluation of Indigenous ways of knowing |  |  |  |  |  |  |
| Fornssler 2018^51^ |  |  | Alternating between one's personal understanding and those of others to acquire new perspectives, clarity, and insight |  |  |  | Weaving local Indigenous knowledge with Western scientific knowledge, while ensuring the integrity of Indigenous knowledge and methodologies |  | Take seriously the commitments, responsibilities, and potential of Indigenous-governed research |  |
| Gray 2019^52^ |  | For the benefit of all |  |  |  |  | Merge science and Indigenous knowledge  See the strengths of Indigenous and Western knowledges |  |  |  |
| Hall 2015a^53^ |  |  |  | Two Row Wampum coming together from distinct world views |  |  | Reflexively weave back and forth   Integrate and connects the best of Indigenous and Western knowledge systems |  | Recognize the need to decolonize across disciplines and bring our own stories into knowledge construction  Decolonizing methodology with a different balancing between Western scientific and Indigenous scientific approaches |  |
| Hall 2015b^54^ |  |  | A learning process and not a perfected outcome | Support renewal of Indigenous ties to culture, land, and language  Contribute to Indigenous cultural renewal |  |  | See the strengths of Indigenous and Western ways of knowing |  | Align with decolonizing and Indigenous research methodologies, governance, and self-determination |  |
| Hatala 2017^55^ |  |  |  | Recognize the benefits of seeing with the strengths of Indigenous and Western ways of knowing |  |  | Indigenous and Western worldviews work alongside one another  Means to bridge Western science and Indigenous knowledge  Using both ways of seeing simultaneously |  |  |  |
| Hatala 2020^56^ |  |  |  |  |  |  | Indigenous and Western worldviews work alongside one another  Means to bridge Western science and Indigenous knowledge |  |  |  |
| Heath-Engel 2016^57^ |  |  |  | Appreciate the multiplicity of perspectives   Recognize and appreciate all knowledges in their own right and entirety  Value diversity and information from individual life journeys  Diversity is key to health  Diverse approaches to health exist within human society |  |  |  |  |  | Two eyes must work together, and all parts of ecosystems must work in harmony to remain healthy  Health is not possible without balance in the cycles of life |
| Hinds 2014^58^ |  | Not just for Indigenous people but all peoples |  | Respect a combination of worldviews with a concerted effort to honour Indigenous paradigms  Normalize Indigenous knowledge |  |  | Combination or blending of theories |  |  | Indigenous and Western worlds are inevitably connected  Sometimes unrealistic to find commonalities between two worlds |
| Hovey 2017^59^ | About life and does not fit into any particular subject area or discipline |  | Gain new understanding (and not necessarily consensus) through the process of conversation | Recognize the benefits of seeing beyond the single perspectives | Always look for another perspective and better ways | Superficial knowledge of other perspectives leads to partial understanding | Bring together perspectives to share a common understanding | Spirit is essential for a complete person |  | Involve a relational process that generates new ideas  A complex relational interaction among people |
| Hunt 2018^60^ |  |  |  |  |  |  | Strengths of Indigenous and western scientific ways of knowing are brought together  Draw upon Indigenous ways of knowing integrated with western science |  |  |  |
| Hutt-MacLeod 2019^16^ |  | Use both perspectives (Indigenous and Western ways of knowing) for the benefit of those being served | Ability to learn to take on other perspective | No single perspective |  |  | Complementary blending of Indigenous and Western methodologies  Embodiment of both Indigenous and Western worldviews  See from one eye the strengths of Indigenous ways of knowing, and from the other eye, the strengths of Western ways of knowing, and to use both perspectives |  |  |  |
| Knapp 2013^61^ |  |  |  | Understand the world from two cultural perspectives |  |  | Bring together and use the strengths of Indigenous and Western ways of knowing |  |  |  |
| Kurtz 2017^62^ |  |  | Illuminate the importance of active and equitable roles in community–academic partner engagement and co-development throughout the research process | Indigenous knowledge, voice, and representation and Western voice are core to research |  |  | Indigenous and Western perspectives |  |  |  |
| Latimer 2014^63^ |  |  |  | Recognize the overlap between two distinct yet evolving knowledge systems |  | No single perspective is ever complete or superior | Embody the best of Indigenous and Western worldviews |  |  |  |
| Latimer 2018^64^ |  |  | Co-learning and integrative process |  |  |  |  |  | Attestation of leadership in research |  |
| Lemke 2017^65^ |  | Building relationships for the benefit of all | Enabling the building of relationships among researchers and Indigenous Peoples | Progressive way forward and a vision to overcome divides between different worldviews  Grounded in the assumption of a need for relationships of trust and respect  Honouring Western and indigenous ways of knowing  Based on the core principles of respect, responsibility, and relationships | Shape the conduct of research and enable cocreation of knowledge |  |  |  |  |  |
| Manmohan 2018^66^ |  |  |  | Allow for better management of the tension between traditional and mainstream approaches to leadership  The gift of multiple perspectives | TES is flexible | Recognize Indigenous knowledge as distinct and whole | Guide conscious decisions to activate the right lens given the circumstances  Weaving of reflexive mainstream and traditional lenses | Awaken the spirit within you   Knowledge is gained from the interaction of body, mind, soul and spirit with all aspects of nature | Align with the decolonizing goal of Indigenous research methodologies | Require becoming observant of the natural world   A ‘coming to know’ process that results from human experience in the natural world |
| Mantyka-Pringle 2017^67^ |  | For the benefit of all |  | Respect differences |  | Using both eyes together gives a new improved understanding | See the strengths of traditional and scientific knowledge |  |  |  |
| Marsh 2015a^68^ |  | Encourage the use of both eyes for the benefit of all |  | Recognize the need for both Western and Indigenous ways of knowing |  | Recognize Indigenous knowledge as distinct and whole | Blending of Aboriginal and Western research methods, knowledge translation, and programme development  Bring together different ways of knowing in a respectful and passionate way |  |  |  |
| Marsh 2015b^17^ |  |  | Encourage development of mutual cultural respect  Encourage authenticity in our relations, both personal and in the research process | Provides an inclusive philosophical, theoretical, and methodological approach  Recognize Indigenous knowledge as a distinct epistemological system co-existing next to mainstream (Western) science  Advocates for inclusion, trust, respect, collaboration, understanding, and acceptance of the strengths that reside in both Western and Aboriginal worldviews  Both worldviews are acknowledged as beneficial |  |  | Blending of Aboriginal and Western research methods, knowledge translation, and program development | Requires an understanding of Indigenous epistemologies, which embody the cosmologies, values, cultural beliefs, and relationships that can vary from one community to another | TES Indigenous decolonizing methodology | Knowledge is relational, shared with creation, and deeply rooted in everything  Researchers are part of the creation of knowledge and the transformation taking place in the research process |
| Marsh 2016^18^ |  |  | Encourage development of mutual cultural respect | Recognize Indigenous knowledge as a distinct and whole knowledge system that exists next to mainstream (Western) science  Incorporate inclusion, humility, honesty, trust, respect, collaboration, understanding, and acceptance of the strengths that reside in both Western and Indigenous worldviews  Both worldviews are acknowledged as beneficial |  | Indigenous knowledge as a distinct and whole knowledge system | Blending of Indigenous and Western research methods, knowledge translation, and program development |  | Example of the application of an Indigenous decolonizing lens to contemporary research |  |
| Marsh 2018^69^ |  |  |  |  |  |  |  |  | Aligns with decolonizing and Indigenous research methodologies  Consistent with Aboriginal governance, research as ceremony, and self-determination  Consistent with the principles of ownership, control, access, and possession |  |
| Martin 2012^70^ | Apply to life itself and does not offer methodologies | Motivate people to leave the world a better place and not compromise for youth  Share knowledge for the greater good  Indigenous and non-Indigenous alike |  | Accept diverse perspectives and employ the most beneficial solutions  Acknowledge and respect a diversity of perspectives without perpetuating dominance  Be mindful of multiple epistemologies and value differences over integration  Diverse knowledge work together to answer most pressing questions | Perspectives of the world are constantly shifting and changing | Each way to see the world is always partial and needs constant reflection | Bring together different ways of knowing  Explain how different types of knowing can be brought together and why they are important | Challenge us to include spiritual and emotional wisdom for a complete understanding of our world |  |  |
| Martin 2009^71^ | Offer guiding principles for all people to understand the world in which we live | We all need one another for knowledge to continue to be produced anew | Requires constant reflection and learning | Different ways of seeing and understanding the world  Does not replicate the dichotomies of a Western science /Indigenous knowledge continuum  Be mind of alternative ways of knowing (multiple epistemologies)   Embrace diverse understandings of reality  Indigenous knowledge and science can both be embraced as legitimate ‘ways of knowing’ | Our perspectives of the world are constantly shifting and changing | Gain a different (not necessarily ‘right’ or better) perspective when seeing through both eyes  Constantly question and reflect on the partiality of one’s perspective  Each eye represents a way to see the world that is always partial | Respect the value of difference over integration or melding of diverse perspectives   Differing perspectives must be reflexively considered  Must not allow any one perspective to dominate or overshadow the other |  |  |  |
| Martin 2017^72^ | Encourages self-reflection to pose questions and critically consider the partiality of one’s perspective |  |  | Value and uses Indigenous and Western ways of knowing  Gift of multiple perspectives  Does not subsume one way of knowing over another; multiple perspectives  Encourage fluidity and multiple perspectives | Encourage fluidity | Self-reflection to pose questions and critically consider the partiality of one’s perspective | See from one eye with the strengths of Indigenous ways of knowing, and to see from the other eye with the strengths of Western ways of knowing, and to use both of these eyes together, for the benefit of all |  |  |  |
| McKivett 2019^74^ |  |  |  |  |  |  | Utilize the strength of both knowledges to create new understandings |  |  |  |
| McKivett 2020^73^ |  |  | A spirit of co-location and collaboration that promotes reciprocity: a give-and- take in the learning process, and a fair distribution of power | Embody acceptance and inclusion to promote mutual cultural respect  Embrace differences as platforms for discussion and innovation |  |  | Reconcile the conflict between Indigenous worldviews and mainstream perspectives in the medical discipline |  | Promote the reclamation of Indigenous rights and ways of being while affirming Indigenous governance over the research process |  |
| McMillan 2016^75^ |  | Scrutinized and balanced for a better vision for tomorrow  Beneficial actions for the next generation | Co-learning journey in which two paradigms will be scrutinized  Produce a common ground for co-existence and co-learning | Respect difference and help breakdown compartmentali-zation of knowledge |  |  | Idealized as the best of both worlds  Respect and balance the energies of two ways  Delineate a cooperative strategy of integration by taking the best of both Western and Indigenous worldviews |  | A decolonizing approach for knowledge creation, mobilization and translation | Sustain ourselves and do not jeopardize the ecological integrity |
| Peltier 2018^78^ |  |  | A process of co-learning |  |  |  | Use the strengths of both eyes to reconcile the use of Western method and theory with Indigenous knowledge |  |  |  |
| Pictou Landing Native Women's Group 2016^44^ |  | For the benefit of all |  |  |  |  | Bring Indigenous and Western knowledge systems together  See the strengths of Indigenous and Western knowledges |  |  |  |
| Rand 2016^79^ |  |  |  |  |  |  | Guiding principle for bringing together multiple worldviews |  |  |  |
| Rowan 2015^76^ |  |  |  |  |  | Engage overlapping perspectives to enjoy a wider and deeper view | Draw together the strengths of knowledges  Dance and weave back and forth between worldviews  Connect the best of Indigenous and Western knowledge systems |  |  |  |
| Rowett 2018^77^ | Way of living life and not limited to a specific subject area or discipline | Motivate people to leave the world a better place and not compromise for youth   For the good of all | Openness towards learning about different perspectives | Does not assimilate Indigenous wisdom into Western knowledge  One way of seeing never dominates the other  Embrace diverse understandings of reality |  | Create a new way of seeing the world that respects differences | Bring together different ways of knowing   Bring teachings together for the good of all  Mindfully utilize both eyes |  | Decolonized approach to research | A method to “link” knowledge systems and explore differences |
| Sasakamoose 2017^80^ |  |  |  | Grappling with each other’s cognitive universes and learning to see through the minds of others is the work of generations to come |  |  | Consider the strengths of Indigenous and Western approaches  Make conscious decisions to activate the more appropriate lens to use or a harmonization of both |  |  |  |
| Shrivastava 2020^81^ |  |  |  | Helped to understand the cultural aspect of integrated oral health care |  |  |  |  |  |  |
| Sivertsen 2020^82^ |  | Use both eyes together for the benefit of all |  | Gift of multiple perspectives |  |  | Aboriginal and Western world views of health and illness need to come together and collaborate  See the strengths of Aboriginal and Western knowledges |  |  |  |
| Sylliboy 2020^83^ |  |  | Integrate the strengths of Indigenous and Western knowledge as a shared-learning process | Does not compromise the essence of the Mi’kmaq knowledge system  Expand and demystifies Indigenous knowledge with other knowledge systems |  |  | Implement the strengths of both knowledge systems   Co-existing without compromising the strengths of each knowledge system |  |  | Incorporate the understanding of the relational as a collective value of kinship  Guide us toward a relational way of being |
| Venner 2018^84^ |  |  |  |  |  |  | An Indigenous process of adaptation that attempts to integrate both Indigenous and Western worldviews to address health concerns |  |  |  |
| Victor 2019^85^ |  |  | Diversity of perspectives leads organically to TES practice  Analysis of data was an opportunity for co-learning |  |  |  |  |  |  |  |
| Vukic 2012^19^ |  |  |  | Promotes a common ground by acknowledging and respecting different worldviews | Indigenous knowledge systems should not be reduced to static categories |  | Honour the blending of Aboriginal and Western understandings of moral governance |  |  |  |
| Vukic 2014^86^ |  | Leave the world a better place and not compromise the opportunities for our youth |  | Bring to the forefront different ways of knowing and understanding the world |  |  | Indigenous knowledge along with Western knowledge can generate knowledge that is meaningful and relevant to Indigenous communities |  |  |  |
| Vukic 2016^87^ |  |  |  | A model to view the world through the lens of western and indigenous knowledge systems |  |  |  |  |  |  |
| Webkamigad 2020^88^ |  |  |  | Understand, acknowledge, and respect a diversity of perspectives of the world |  |  | Does not perpetuate the dominance of one perspective over the other |  |  |  |
| Whiting 2018^89^ |  |  |  | Establish common ground and respect of different worldviews |  |  | Incorporating or ‘‘weaving’’ of traditional and mainstream knowledges |  |  |  |
| Whitty-Rogers 2013^8^ |  |  |  |  |  |  | Blend scientific and medical knowledge that considers Aboriginal cultures  Blend Western and Indigenous knowledges together  Not meant to blend but bring together each other’s different ways of knowing to understand one another |  |  |  |
| Whitty-Rogers 2016^9^ |  |  |  |  |  |  | See the strengths of Indigenous and Euro-Western ways of knowing |  |  |  |
| Wolfson 2019^90^ |  |  |  | The alignment of Indigenous and Western knowledges and worldviews as distinct epistemological systems  Respect and integrates the strengths of Indigenous knowledge and Western science |  |  | Weaving back and forth between the two worldviews without assimilation or supremacy  Respect and integrates the strengths of Indigenous knowledge and Western science |  |  |  |
| Wright 2019a^91^ |  | Relational accountability of researchers |  | Advocate viewing the world with Indigenous and Westernized knowledges |  |  | Merging knowledges together is strengths-based. |  | Privilege Indigenous peoples’ knowledge in the research design |  |
| Wright 2019b^93^ |  |  |  |  |  |  | View the world through the best of both mainstream and Indigenous perspectives |  |  |  |
| Wright 2019c^94^ |  |  |  | Including diverse perspectives allows us to better interpret and understand a phenomenon |  |  |  |  |  |  |
| Wright 2019d^95^ |  |  | Involving multiple perspectives within the research process creates new meaning |  |  |  |  |  |  |  |
| Wright 2020^92^ |  |  | TES was engaged throughout data analysis to ensure that team members could add their perspective to the way that data were interpreted |  |  |  |  |  |  |  |
