# Supplementary material for: How is Etuaptmumk/Two-Eyed Seeing characterized in Indigenous health research? A scoping review
Source: PLoS One. 2021 Jul 20;16(7):e0254612. doi: 10.1371/journal.pone.0254612 (PMC8291645; doi:10.1371/journal.pone.0254612)
Supplement: Attachment — Submitted filename: Response to Reviewers.docx [file pone.0254612.s009.docx]

Dear Dr. Sarah Munce,

Thank you for considering our manuscript “How is Etuaptmumk/Two-Eyed Seeing Characterized in Indigenous Health Research? A Scoping Review” for publication and for sending along the editor’s comments. We have addressed the editor’s feedback in the attached revised manuscript. The changes are explained below.

Please note that we have included the following items when submitting our revised manuscript:

- This rebuttal letter, which responds to each point raised by the academic editor and reviewers. We have uploaded this letter as a separate file labeled 'Response to Reviewers'.
- A marked-up copy of the manuscript that highlights changes made to the original version. We uploaded this as a separate file labeled 'Revised Manuscript with Track Changes'.
- An unmarked version of the revised paper without tracked changes. We uploaded this as a separate file labeled 'Manuscript'.

We have provided an itemized list of our responses to the editor’s comments below:

1. Abstract. In the abstract, please write out the Joanna Briggs Institute rather than use the acronym JBI, which has not been previously introduced.

Response: Thank you for your comment. One of our co-authors (Ziwa Yu) is a JBI-certified reviewer. She was notified in Jan 21, 2021 from JBI’s Global Engagement Administration Officer that JBI went through a rebranding process and discontinued the use of Joanna Briggs Institute. They specifically requested that we use only the term ‘JBI’ when referring to the research organization.

2. Conclusion
   Please remove the sentence: “Once again, the purpose of highlighting these differences is not to judge or criticize, but to encourage a larger dialogue about Two-Eyed Seeing and the impact of language on how the guiding principle is understood”.

Response: We removed the sentence.

3. Rather than “This reflection is timely…” please replace with “This review is timely …”

Response: We made this change.

4. Please remove the sentence: “We hope that this review will assist aspiring researchers in their learning journey”.

Response: We removed the sentence.

5. While revising your submission, please upload your figure files to the Preflight Analysis and Conversion Engine (PACE) digital diagnostic tool, <https://pacev2.apexcovantage.com/>. PACE helps ensure that figures meet PLOS requirements. To use PACE, you must first register as a user. Registration is free. Then, login and navigate to the UPLOAD tab, where you will find detailed instructions on how to use the tool. If you encounter any issues or have any questions when using PACE, please email PLOS at [figures@plos.org](mailto:figures@plos.org). Please note that Supporting Information files do not need this step.

Response: We uploaded the figures to PACE, which confirmed that they were in the correct format.
